# Heterogeneity of Cellular Senescence, Senotyping, and Targeting by Senolytics and Senomorphics in Lung Diseases

**DOI:** 10.3390/ijms26199687

**Published:** 2025-10-04

**Authors:** Said Ali Ozdemir, Md Imam Faizan, Gagandeep Kaur, Sadiya Bi Shaikh, Khursheed Ul Islam, Irfan Rahman

**Affiliations:** Department of Environmental Medicine, University of Rochester Medical Center, Rochester, NY 14642, USA; saidali_ozdemir@urmc.rochester.edu (S.A.O.); mdimam_faizan@urmc.rochester.edu (M.I.F.); gagandeep_kaur@urmc.rochester.edu (G.K.); sadiya_shaikh@urmc.rochester.edu (S.B.S.); khursheedulislam_ganaie@urmc.rochester.edu (K.U.I.)

**Keywords:** cellular senescence, senolytics, senomorphics, aging, age related diseases, senotherapeutics, cellular heterogeneity

## Abstract

Cellular senescence, a state of stable cell cycle arrest accompanied by a complex senescence-associated secretory phenotype (SASP), is a fundamental biological process implicated as a key driver of lung aging and lung age-related diseases (LARDs). This review provides a comprehensive overview of the rapidly evolving field of senotyping based on cellular heterogeneity in lung development and aging in health and disease. It also delves into the molecular mechanisms driving senescence and SASP production, highlighting pathways such as p53/p21, p16^INK4a^/RB, mTOR, and p38 MAPK as therapeutic targets. The involvement of various novel SASP proteins, such as GDP15, cytokines/chemokines, growth factors, and DNA damage response proteins. We further highlight the effectiveness of senotherapeutics in mitigating the detrimental effects of senescent cell (SnC) accumulation within the lungs. It also outlines two main therapeutic approaches: senolytics, which selectively trigger apoptosis in SnCs, and senomorphics (also known as senostatics), which mitigate the detrimental effects of the SASP without necessarily removing the senescent cells. Various classes of senolytic and senomorphic drugs are currently in clinical trials including natural products (e.g., quercetin, fisetin, resveratrol) and repurposed drugs (e.g., dasatinib, navitoclax, metformin, rapamycin) that has demonstrated therapeutic promise in improving tissue function, alleviating LARDs, and extending health span. We discuss the future of these strategies in lung research and further elaborate upon the usability of novel approaches including HSP90 inhibitors, senolytic CAR-T cells, Antibody drug conjugate and galactose-modified prodrugs in influencing the field of personalized medicine in future. Overall, this comprehensive review highlights the progress made so far and the challenges faced in the field of cellular senescence including SnC heterogeneity, states of senescence, senotyping, immunosenescence, drug delivery, target specificity, long-term safety, and the need for robust cell-based biomarkers. Future perspectives, such as advanced delivery systems, and combination therapies, are considered critical for translating the potential of senotherapeutics into effective clinical applications for age-related pulmonary diseases/conditions.

## 1. Introduction

Cellular senescence is defined as a stable and often irreversible arrest of the cell cycle, induced by a variety of stressors such as telomere attrition (replicative senescence), DNA damage, oncogenic signaling, and oxidative stress [1,2,3]. Hallmark features of senescent cells include upregulation of cyclin-dependent kinase inhibitors such as p16^Ink4a^ and p21^Cip1/Waf1^, resistance to apoptosis, altered nuclear morphology, increased activity of senescence-associated β-galactosidase (SA-β-gal) and development of a complex secretory profile known as the senescence-associated secretory phenotype (SASP) [1,2,4,5]. The SASP includes a broad range of secreted factors, such as pro-inflammatory cytokines (e.g., IL-6, IL-8), chemokines, growth factors, and matrix metalloproteinases (MMPs) [2,3,6,7]. Its composition and functional consequences are highly context-dependent, determined by the senescence-inducing stimulus, cell type heterogeneity, and local tissue microenvironment [7,8].

Cellular senescence is a heterogeneous process encompassing multiple distinct subtypes, each initiated by specific stimuli and governed by unique molecular mechanisms. Replicative senescence (RS) represents a classical form of permanent cell cycle arrest that arises after repeated cellular divisions. This process is primarily driven by progressive telomere shortening, which, upon reaching a critical length, is perceived as persistent DNA damage. The subsequent activation of the DNA damage response (DDR) engages key tumor suppressor pathways, particularly the p53/p21^Cip1/Waf1^and p16^Ink4a^/retinoblastoma (Rb) axes, resulting in stable growth arrest and adoption of the senescent phenotype [9,10]. This mechanism is particularly relevant in the aging lung, where the accumulation of senescent alveolar epithelial cells (AECs) disrupts alveolar integrity and impairs tissue repair [11].

Stress-induced premature senescence (SIPS) is driven by exogenous stressors, such as cigarette smoke, reactive oxygen species (ROS, and persistent inflammation that are characteristic of chronic lung diseases such as chronic obstructive pulmonary disease (COPD) and idiopathic pulmonary fibrosis (IPF) [12,13,14]. Oncogene-induced senescence (OIS) constitutes another critical subtype, driven by hyperactivation of oncogenes such as RAS or BRAF. This leads to replication stress and a robust DDR, which initially serves as a tumor-suppressive barrier [15]. However, in chronic contexts, the associated SASP may paradoxically facilitate tumor progression, particularly in the early stages of lung adenocarcinoma [6,16].

Therapy-induced senescence (TIS) has gained increasing recognition in the lungs of cancer survivors, where exposure to chemotherapeutic agents or thoracic radiation initiates senescence pathways that contribute to fibrosis and impaired regenerative capacity [17,18,19]. A non-canonical form, mitochondrial dysfunction-associated senescence (MiDAS), emerges from mitochondrial stress and altered metabolic homeostasis rather than direct DNA damage. This variant is characterized by disrupted NAD^+^/NADH ratios, activation of AMP-activated protein kinase (AMPK), and engagement of p53 signaling, often in the absence of elevated ROS, and exhibits a distinct SASP signature. MiDAS has been implicated in age-associated lung pathologies linked to mitochondrial decline [20].

Importantly, cellular senescence also plays physiological roles; for instance, developmental senescence by transiently activating p21, transient oncogene-induced senescence during embryogenesis contributes to organ patterning via p21^Cip1/Waf1^-dependent but p53-independent mechanisms, without leading to pathological outcomes [21]. Additionally, paracrine, or bystander senescence occurs when SASP components propagate senescent signaling to neighboring non-senescent cells, amplifying local tissue dysfunction and senescence burden [22,23]. Further complicating the landscape is the concept of pseudosenescence, which is a reversible state in which cells exhibit certain senescence markers without undergoing complete cell cycle arrest posing challenges for biomarker interpretation and therapeutic precision [24,25].

Cellular senescence plays a paradoxical role in tissue homeostasis, acting as both a protective and pathological mechanism. Under physiological conditions, senescence serves to suppress the proliferation of damaged, stressed, or oncogene-activated cells, thereby preserving genomic stability and preventing malignant transformation [26]. The lungs, continuously exposed to environmental insults, such as cigarette smoke, particulate matter, ozone, pathogens, and occupational irritants, are particularly vulnerable to cellular damage and premature aging. These exposures elicit oxidative stress, DNA damage, mitochondrial dysfunction, and chronic inflammation all well-established inducers of cellular senescence [27,28,29,30,31]. In normal lung development and repair, transient senescence is functionally beneficial. Senescent cells contribute to wound healing and regeneration by secreting growth factors and remodeling components of the extracellular matrix (ECM) via the SASPs [21,32,33,34]. However, persistent accumulation of senescent cells particularly in post-mitotic tissues like the lung leads to homeostatic disruption. Chronically active SASP secretion by senescent alveolar epithelial cells, endothelial cells, and fibroblasts promotes sustained inflammation, matrix stiffening, and immune dysregulation, thereby impairing regenerative capacity and advancing diseases, such as IPF and COPD [35,36].

Briefly, in context with lung diseases, chronic environmental exposure also induces stress-induced premature senescence (SIPS) in both epithelial and stromal lung cells [29,37], leading to persistent SASP signaling that fuels inflammation, hinders tissue repair, and promotes fibrotic remodeling, particularly in COPD and IPF [38,39]. In COPD, cigarette smoke triggers senescence in airway epithelial cells and fibroblasts, resulting in a pro-inflammatory phenotype characterized by the secretion of cytokines such as IL-6, IL-8, and MMPs, which degrade the ECM and sustain chronic inflammation [38,40]. Senescent endothelial cells also contribute to vascular remodeling and emphysematous changes in COPD lungs [38]. In IPF, senescence of alveolar type II (AT2) cells is driven by telomere dysfunction, inherited mutations (e.g., in TERT or RTEL1), and environmental stressors. These senescent AT2 cells lose regenerative potential and produce SASP components that activate immune cells and fibroblasts, thereby fostering fibrogenesis and inhibiting epithelial repair [11,41]. Senescence also intersects with tumorigenesis. OIS acts as an early tumor-suppressive mechanism; however, chronic SASP signaling can create a permissive tumor microenvironment that facilitates immune evasion and promotes malignant progression [6,42]. More recently, senescence has been implicated in acute lung injury (ALI) and COVID-19-associated lung pathology. In these contexts, viral infection and inflammation provoke transient senescence in epithelial cells, which impairs resolution and contributes to fibrosis and long-term pulmonary dysfunction [43,44]. Collectively, emerging evidence underscores that the chronic accumulation of senescent cells in the lung disrupts tissue homeostasis, amplifies inflammatory signaling, and impairs regenerative capacity positioning senescence not merely as a consequence of disease, but as a central driver of pulmonary pathology [35]. This paradigm shift has catalyzed growing interest in senotherapeutics, including senolytics agents that selectively eliminate senescent cells and senomorphics, which suppress the deleterious effects of the SASP and other mediators [35,45].

**Figure 1:**
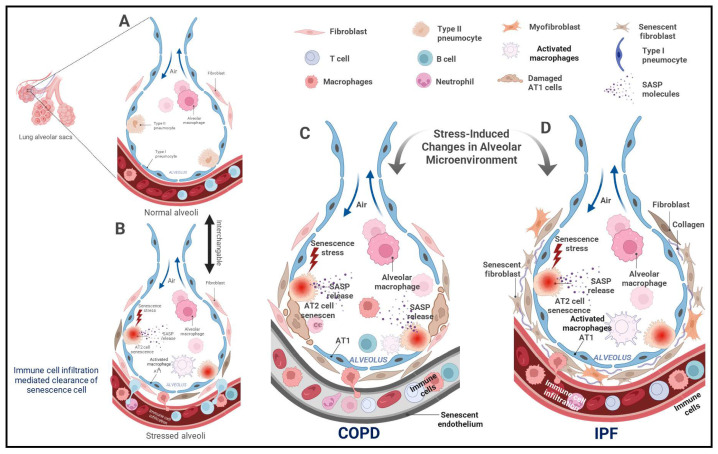
Schematic representation of alveolar alterations during normal, reversible, and irreversible lung damage in COPD and IPF progression. Panels (**A**,**B**) depict healthy alveolar architecture and a moderately stressed state, respectively. Under mild injury, immune cell infiltration may support tissue repair, allowing for reversibility. In contrast, panels (**C**,**D**) illustrate irreversible damage observed in advanced COPD and IPF, respectively. COPD progression is characterized by alveolar epithelial cell loss and emphysematous changes driven by SASP-associated senescence and endothelial dysfunction. In IPF, fibroblast accumulation and excessive collagen deposition dominate the pathology. Addressing both epithelial damage and fibrotic remodeling remains a significant therapeutic challenge in chronic lung diseases.

Recent findings suggest that cellular senescence in lung disease extends well beyond epithelial cell cycle arrest. Senescence affects a broad spectrum of cell types including fibroblasts, endothelial cells, immune cells, and alveolar type I and II (AT1 and AT2) epithelial cells (Figure 1), each contributing uniquely to the progression of inflammation, matrix remodeling, and failure of tissue repair [46]. Notably, senescent type II alveolar epithelial cells (AEC2s) lose their progenitor function, further compromising alveolar integrity and repair [11]. The inherently slow turnover of distal alveolar regions exacerbates this vulnerability, as insufficient regenerative input allows the accumulation of damaged or senescent cells over time [47,48,49,50]. However, what is lacking is the ability to map the senescent cells at various stages of senescence progression (early, intermediate and late stage) and delineating the importance of their accumulation in the spatial context within the aged/diseased tissue [51,52,53]. Furthermore, a substantial heterogeneity exists within senescent cell populations, leading to variable SASP profiles and regenerative capacity. The spatial and functional diversity of these senescent subsets across lung compartments remains incompletely characterized [51,52,53].

Furthermore, metabolic reprogramming and mitochondrial dysfunction particularly in AT2 cells have been implicated in reinforcing fibrogenic signaling and destabilizing epithelial integrity, though these pathways require deeper mechanistic insight [20,54,55,56]. Challenging the traditional view of senescence as irreversible, recent studies indicate that senescent phenotypes may be reversible in certain lung cell types, particularly under the influence of pharmacological agents or microenvironmental cues, opening new therapeutic possibilities [57]. Underexplored signaling networks, such as the YAP/TAZ-driven mechano-transduction axis, activated by ECM stiffening, have been shown to reinforce fibroblast senescence and activation in fibrotic lung tissue, and merit further investigation [58]. Additionally, persistent senescence may be sustained by impaired immune surveillance, particularly due to aging-associated dysfunction in senescent cell clearance mechanisms [59]. The potential role of the lung microbiome in modulating cellular senescence and SASP expression remains largely speculative but could significantly influence disease trajectory [60].

Taken together, these underexplored dimensions reframe cellular senescence as a dynamic, multicellular process central to lung disease pathogenesis and therapeutic intervention. This comprehensive review aims to provide a focused analysis of these emerging themes to facilitate a better understanding of senescence based on cellular heterogeneity, senotypes, and senotherapy in pulmonary disorders.

## 2. Molecular Mechanisms of Cellular Senescence

Here, we briefly summarize the molecular mechanisms underlaying the induction and maintenance of cellular senescence. The induction and maintenance of cellular senescence are orchestrated by a complex network of molecular pathways activated in response to diverse intrinsic and extrinsic stressors, including telomere attrition, DNA damage, and oncogenic signaling [1,2,3]. Such stresses signals ultimately converge on tumor suppressor networks that enforce a durable cell cycle arrest [1,2]. Among these, the DDR represents a central and historically pivotal mechanism, particularly in the theme of replicative senescence, where progressive telomere shortening activates the DDR and triggers downstream effectors [10,61]. This response primarily engages two key tumor suppressor pathways: the p53/p21^Cip1/Waf1^ axis and the p16^Ink4a^/ Rb pathway. Together, these signaling cascades reinforce and stabilize the senescent phenotype by halting cell cycle progression and preventing proliferation of damaged cells [10,61]. In addition to canonical DDR-mediated senescence, alternative mechanisms such as mitochondrial dysfunction, epigenetic regulation have also been implicated. Specifically, metabolic stress, histone modifications, and imbalances in NAD^+^/NADH ratios can induce a senescence program characterized by altered energy metabolism and a distinct SASP profile [20,62,63].

### 2.1. DNA Damage Response (DDR) and Telomere Attrition

The DDR constitutes a central mechanism in the induction of cellular senescence [2,3,10]. Critically shortened telomeres are perceived as persistent, irreparable DNA lesions, thereby initiating a robust DDR that enforces a stable and durable growth arrest [9,10,27]. This cascade converges on key tumor suppressor pathways, most notably the activation of p53, which subsequently induces the expression of the cyclin-dependent kinase inhibitor p21^Cip1/Waf1^ [2,3,4,64]. p21^Cip1/Waf1^ inhibits CDK2 activity, leading to hypo-phosphorylation of the RB protein and resulting in irreversible cell cycle exit [2,15,64]. In parallel, DDR signaling induces significant chromatin remodeling, including the formation of senescence-associated heterochromatin foci (SAHF) and DNA segments with chromatin alterations reinforcing senescence (DNA-SCARS), both of which contribute to the long-term maintenance of the senescent state [1,2,3,64]. Importantly, DDR activation is not limited to telomere attrition. Other stressors, such as oncogene activation which induces replication stress and exposure to genotoxic agents, can similarly initiate DDR-driven senescence [2,3,6,15,64,65].

### 2.2. Mitochondrial Dysfunction and Oxidative Stress

Mitochondrial dysfunction-mediated ROS generation is a well-established driver of cellular senescence in the lung [1,2,3], and one of the primary triggers of stress-induced premature senescence (SIPS) in chronic lung diseases, including COPD and IPF. The lungs are continually exposed to environmental stressors such as cigarette smoke and airborne pollutants, which induce substantial oxidative stress [38,66]. Elevated ROS levels cause oxidative DNA damage, which in turn activates canonical senescence pathways in various lung cell types [67,68].

In COPD, for instance, senescent small airway fibroblasts exhibit pronounced mitochondrial dysfunction, along with transcriptional signatures indicative of oxidative stress and impaired mitochondrial bioenergetics [69]. Beyond ROS-mediated mechanisms, senescence can also be initiated through a distinct, non-canonical pathway including termed MiDAS. This form of senescence is not dependent on DNA damage or high ROS levels but is instead driven by metabolic perturbations such as altered NAD^+^/NADH ratios and activation of AMPK [55,70,71]. MiDAS has been implicated in age-related lung diseases characterized by progressive mitochondrial decline, contributing to fibrogenic signaling and epithelial instability, particularly within AT2 cells [55,70,71]. Together, both ROS-dependent and ROS-independent mitochondrial pathways represent critical mechanisms underlying the accumulation of senescent cells in lung pathobiology.

### 2.3. Epigenetic Regulation of Senescence in Lung Cells

Epigenetic alterations play a central role in the initiation and maintenance of the senescent state in lung cells and are considered integral to the “hallmarks of aging” [8,65,72]. One of the defining features of senescent cells is extensive chromatin reorganization, including the formation of senescence-associated hetero-chromatin foci (SAHF) which silence proliferation-promoting genes), and the emergence of DNA-SCARS [1,2,3,64], thus reinforcing senescence DNA-SCARS. These structural changes are accompanied by global modifications of histones and the downregulation of key nuclear lamina components, such as Lamin B1, which collectively stabilize the senescent cell cycle arrest [1,2,3,64].

Recent findings have highlighted that specific molecular events such as the degradation of histone deacetylase 4 (HDAC4) can initiate an enhancer-based epigenetic program driven by AP-1 and p300, contributing directly to the senescent phenotype [64]. Other HDACs1-3 may also participate in a similar senescence phenotype via their reduction in a co-repressor complex [73]. In the context of lung disease, epigenetic dysregulation emerges as a prominent and pathogenic feature. In COPD, for instance, accelerated lung aging is associated with distinct alterations in DNA methylation and epigenetic clocks such as DNAmGrimAge have been strongly correlated with disease risk and severity [71]. Moreover, epigenetic biomarkers of aging have shown strong associations with lung function decline in elderly populations [74,75]. Dysregulation of key epigenetic regulators, such as the NAD^+^-dependent histone deacetylase Sirtuin 1(SIRT1), is also observed in immune cells from COPD patients and has been linked to persistent inflammation and impaired resolution [76]. These findings emphasize that epigenetic mechanisms in the lung not only serve as markers of biological aging, but also act as active drivers of cellular senescence, offering attractive targets for therapeutic intervention in age-associated pulmonary diseases [77]. Yet the area of epigenetic regulation of senescence remains greatly underexplored with little understanding of the chromatin organization and transcriptional regulation in various cell types, an area that warrants further research.

### 2.4. Biological States of Cellular Senescence

Senescence is not a single endpoint but exists in diverse states, shaped by the initiating stress, cell type, and tissue context. The replicative state arises from telomere erosion [78,79], whereas stress-induced premature senescence is triggered by DNA damage, oncogenic signaling, oxidative stress, or mitochondrial dysfunction [20,23,80,81]. A distinct p16^INK4a^-mediated state is frequently observed in aging tissues and stem-cell niches, where elevated p16 drives a stable growth arrest independent of telomere length, contributing strongly to organismal aging [18,82,83,84]. Another developmental state occurs transiently during embryogenesis and remodeling [21,85], while acute states aid tissue repair and chronic states promote persistent inflammation and fibrosis [41,86,87,88,89,90,91]. Tracking these states is challenging, as no single marker universally defines senescence. To address this, senotyping frameworks classify cells by initiating stimuli, transcriptomic signatures, and functional outputs. For example, replicative senescence can be tracked by telomere attrition and persistent p^53/p21^ activity [92,93,94], whereas SASP-based senotyping distinguishes SASP-high inflammatory fibroblasts from SASP-low or metabolic states [36,95]. Other categories include mitochondrial-dysfunction–associated senescence (MiDAS) and DNA-damage dominant states defined by multi-omics profiling [20,80]. Yet, many cells remain unclassified or hybrid, reflecting program plasticity [96,97,98,99]. Additionally, senescent cells may transition between stable states with active SASP and deep states marked by chromatin remodeling and metabolic decline [9,73,100,101,102]. These states may be dependent on the specific hallmarks of aging, cellular proliferation state, immune-senescence state, epigenetic state, and secretory state. Collectively, senescence emerges as a continuum of states, where senotyping provides a framework to resolve heterogeneity and link senotypes to functional outcomes.

## 3. The Dichotomous Role of Senescence in Lung Health and Disease

Cellular senescence exemplifies the principle of antagonistic pleiotropy, exerting both beneficial and deleterious effects that are highly dependent on physiological context and temporal dynamics based on cellular heterogeneity in lung development and diseases [6,8,103].

### 3.1. Beneficial Roles

Transient cellular senescence plays an important role in normal physiological processes. During embryonic development, programmed senescence contributes to tissue patterning and morphogenesis [1,104]. In the context of acute tissue injury, senescent cells support wound healing and limit fibrotic remodeling by secreting reparative factors such as PDGF-AA (Platelet-Derived Growth Factor-AA) and MMPs, which promote tissue repair and recruit immune cells for the clearance of damaged or apoptotic cells [2,6,8,105]. Arguably, the most well-established beneficial function of senescence is tumor suppression. This occurs through both cell-autonomous mechanisms, via stable cell cycle arrest, and non-cell-autonomous mechanisms, through SASP-mediated immune surveillance that facilitates the clearance of potentially malignant cells [1,2,6,106].

### 3.2. Detrimental Roles

In contrast, the chronic accumulation of senescent cells particularly with aging or in the context of unresolved tissue stress has deleterious effects [1,8,103]. This accumulation is exacerbated by immunosenescence, the age-associated decline in immune surveillance capacity, which impairs the effective clearance of senescent cells [1,4]. The resulting persistent SASP induces a chronic, low-grade inflammatory state known as “inflammaging,” a hallmark of aging and numerous age-related disorders [1,8]. This unresolved pro-inflammatory milieu disrupts tissue architecture, promotes paracrine senescence in neighboring cells, depletes tissue-resident stem cell populations, and fosters a microenvironment conducive to tumorigenesis. Consequently, senescence contributes to the pathophysiology of a broad spectrum of diseases, including cancer, type 2 diabetes, cardiovascular disease, IPF, liver fibrosis, osteoarthritis, neurodegenerative disorders such as Alzheimer’s disease, and clinical frailty [1,2,6,8,65,103,104,107,108,109,110]. Understanding the dynamic balance between the beneficial and harmful effects of senescence across tissue types and temporal contexts is critical for designing effective, context-specific therapeutic interventions [2].

## 4. Roles of Cellular Senescence in Lung Diseases: Role of Cellular Senescence in Lung Diseases Based on Cellular Heterogeneity

Cellular senescence is recognized as a key contributor to the onset and progression of several chronic lung diseases, including COPD, IPF, PAH, and asthma [82,111,112]. Senescent cells accumulate in response to stressors such as oxidative damage, telomere shortening, and environmental exposures like cigarette smoke [14,26,29,38,113]. The milieu of the SASP is highly heterogeneous based on heterogeneous nature of senotypes, and is determined by both the originating cell type and the surrounding disease microenvironment.

In fibroblasts, the senescence-associated phenotype includes the secretion of IL-6, IL-8, and MMPs, which promote inflammation, ECM remodeling, and even tumor progression [6,114,115]. In AT2 epithelial cells, particularly in IPF senescence, results in a pro-fibrotic SASP enriched in Transforming Growth Factor Beta (TGF-β) and Plasminogen Activator Inhibitor-1 (PAI-1), contributing to fibrosis and impaired regenerative responses [116]. In COPD, senescent airway epithelial cells secrete IL-6, IL-8, CCL2, and MMP-12, exacerbating chronic inflammation and alveolar destruction [82,117,118,119]. Notably, in post-COVID-19 lung fibrosis, epithelial SASP profiles including IL-1β, CCL2, and TGF-β resemble those observed in IPF [120].

In COPD, bronchial epithelial cells, fibroblasts, and endothelial cells undergo senescence, driving inflammation, alveolar wall thinning, and progressive loss of lung function [121]. In asthma, especially in elderly individuals, senescent airway epithelial and smooth muscle cells accumulate, leading to abnormal tissue remodeling and airway dysfunction [122]. Following severe COVID-19, persistent senescence in AT2 cells, transitional epithelial cells, and endothelial cells has been documented, contributing to fibrosis and recapitulating IPF-like features [112]. Similarly, in radiation-induced lung fibrosis, senescent epithelial, fibroblast, and vascular endothelial cells exhibit stable growth arrest and fail to support effective tissue repair [35,91].

These disease-specific patterns underscore the role of cell type-dependent senescence in lung pathology and emphasize the therapeutic potential of targeting senescence at the cellular level. In pulmonary hypertension and post-COVID complications, senescence of endothelial and epithelial cells further contributes to vascular remodeling and fibrotic progression [123,124,125,126]. Far from being a passive byproduct of aging, cellular senescence actively drives disease processes. As such, it represents a compelling target for novel interventions including senolytics and senomorphics which aim to reduce the burden of senescent cells or modulate the SASP to restore pulmonary homeostasis [127,128].

Across multiple lung diseases, senescence of distinct cell types contributes to pathology through impaired tissue regeneration, persistent inflammation, and structural remodeling [35,90,119]. This section discusses the current understanding of the involvement of senescence in disease onset, progression or exacerbation in the lung. Although direct evidence on age-dependent changes in lung cellular senescence remains limited, a substantial body of literature supports the role of senescence in various pulmonary pathologies, including IPF, PAH, asthma, COPD, and lung cancer. In the following sections, we provide an overview of key senescence markers identified in each of these disease contexts, with a particular focus on their expression within distinct lung cell populations.

### 4.1. Senescence in COPD

**Figure 2:**
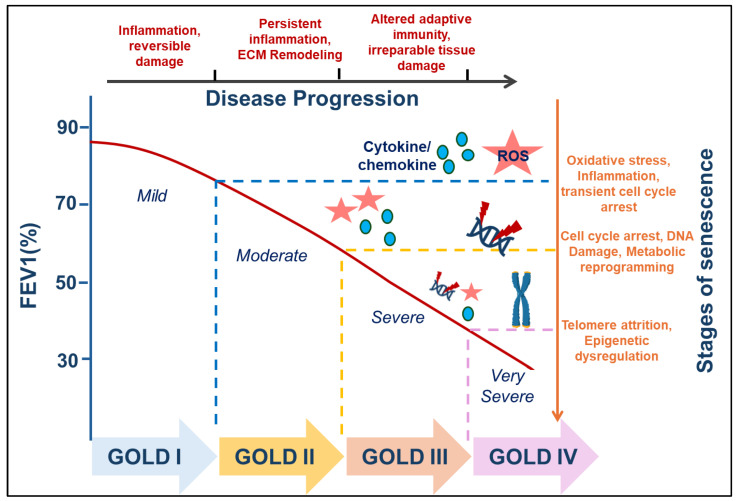
Stage-wise representation of COPD progression and associated senescence markers. The figure illustrates the progression of COPD across GOLD stages 1 to 4 (*x*-axis), with corresponding decline in FEV_1_ (% predicted) depicted along the *y*-axis. The upper trajectory highlights the cumulative tissue damage over time, while the lower segmented arrow represents the GOLD classification of COPD severity, showing an inverse relationship with FEV_1_ levels. As disease severity increases with age-related decline in lung function, there is a concomitant rise in the expression of senescence-associated markers, indicating a potential link between disease progression and cellular senescence.

COPD is a progressive lung disorder predominantly associated with underlying inflammation, alveolar destruction, and aging. Clinically, COPD encompasses phenotypes such as chronic bronchitis and emphysema, with small airway disease being a central pathological feature. The disease is characterized by airflow limitation, and extensive structural remodeling of the airways and lung parenchyma. Accumulating evidence demonstrates increased cellular senescence in key lung cell populations including type II alveolar epithelial cells, endothelial cells, and fibroblasts in emphysematous lungs [29,129]. A meta-analysis involving 6,378 individuals further supported this association, revealing a significant correlation between shortened leukocyte telomere length and increased COPD risk [130] (Figure 2).

Given that cigarette smoke is the primary etiologic factor in COPD, several in vitro studies have investigated its role in epithelial senescence. These studies report upregulation of senescence-associated markers including p21^Cip1/Waf1^, p53, SA-β-Gal, CXCL5, CXCL8, VEGF, and the DNA damage marker Gamma-Histone H2A Variant X (γH2A.X) [119,131]. Early evidence showed the role of p21^Cip1/Waf1^ [132] and later p16^Ink4a^ [13] in regulation of cellular senescence in COPD. However, the role of p16^Ink4a^ remains debated. While murine models show increased p16^Ink4a^ expression in COPD, prior work by Sundar et al. (2018) showed that genetic ablation of p16^Ink4a^ alone did not protect against smoke-induced senescence [13]. Subsequent studies, however, suggest that p16^Ink4a^ contributes to the regulation of smoke-mediated senescence responses [82,133]. Additionally, Woldhuis et al. (2020) demonstrated elevated p16^Ink4a^ expression in COPD-derived fibroblasts, which inversely correlated with decorin expression, linking accelerated senescence with ECM dysregulation [46].

Senescence propagation may also occur through extracellular vehicles (EVs) carrying senescence-promoting microRNAs. In a recent study, EVs from COPD patients were shown to carry miR-34a, which induced senescence in healthy small airway epithelial cells (SAECs) by downregulating SIRT1 and upregulating p21^Cip1/Waf1^, suggesting a mechanism for both local disease exacerbation and systemic comorbidities [134]. Recombinant human CC16 (rhCC16) has demonstrated senescence-reducing effects in lung epithelial cells and COPD mouse models by downregulating senescence markers, such as β-galactosidase, p16^Ink4a^, p21^Cip1/Waf1^, and ROS. These effects are mediated via activation of the AMPK/SIRT1-PGC-1α signaling pathway, thereby restoring mitochondrial function and offering promise as a senescence-modulating therapy [135].

A clinical trial supplementing COPD patient with nicotinamide riboside (NR) a precursor of NAD^+^ reported reduced airway inflammation (IL-8), increased systemic NAD^+^ levels, and improvements in genomic integrity and epigenetic aging, highlighting its therapeutic potential in targeting cellular senescence [136]. Yao et al. showed SIRT1 via FOXO3 mediated pathways attenuate lung cellular senescence in COPD/emphysema [137]. SIRT1 is reduced in lungs of patients with COPD [138]. Bioinformatics studies using machine learning and weighted gene co-expression network analysis (WGCNA) have identified four key genes EP300, mTOR, NFE2L1, and TXN as molecular links between COPD and aging. A diagnostic model based on these genes, developed using artificial neural networks, showed high predictive accuracy for COPD [139]. A six-month physical activity intervention in COPD patients reduced the proportion of senescent T-lymphocytes, improved their proliferative capacity, and reduced their ability to induce fibroblast senescence, thereby improving immune function [140].

Cellular senescence also appears to link COPD and cancer pathogenesis. A 2018 study showed that exposure of human bronchial epithelial cells (HBECs) to serum from COPD patients led to increased markers of senescence- SA-β-Gal, γH2A.X, p21^Cip1/Waf1^, and ROS. The conditioned medium from these cells contained elevated CXCL5, CXCL8/IL-8, and VEGF, which promoted adhesion, proliferation, and migration of hypersecretory cells. These findings suggest that COPD-associated senescence may create a pro-tumorigenic microenvironment, independent of smoking status [119].

A pilot study revealed that induced sputum cells in COPD patients exhibit higher biological aging than peripheral blood leukocytes, as measured by DNA methylation age (DNAmAge) and age acceleration (AgeAcc). While telomere length did not show strong correlation, lung function (FEV_1_%) and use of inhaled corticosteroids were associated with reduced biological aging in leukocytes suggesting a clinical relevance for site-specific aging markers [141]. While the concept of targeting senescence in COPD is compelling, it remains an emerging field requiring further validation. COPD is a highly heterogeneous disease, with variability in clinical manifestations depending on the location of injury, type of insult, patient age, sex, and comorbid conditions. Although senescence is a critical component of accelerated lung aging, it is not the sole driver of COPD pathogenesis. Importantly, senescence also serves physiological roles in tissue repair and immune regulation. Therefore, indiscriminate elimination of senescent cells may lead to adverse effects. A more nuanced approach focused on identifying and targeting pathogenic senescent cell subtypes that contribute to irreversible tissue damage, immune dysfunction, and stem cell depletion is essential for developing effective senescence-directed therapies in COPD.

#### Cellular Senescence Across Different COPD Stages and Severities

The role and extent of cellular senescence in COPD appear to vary based on disease severity and clinical phenotype. While systemic factors capable of inducing senescence are present across all stages of COPD, accumulating evidence suggests that the burden of senescent cells in the lung correlates with disease progression and severity (Figure 2).

Studies analyzing patient-derived cells and tissues indicate that the accumulation of senescent cells is more pronounced in severe forms of COPD, particularly in early-onset cases. In a study by Woldhuis et al., fibroblasts isolated from patients with severe, early-onset COPD (SEO-COPD) and those with mild-to-moderate COPD were examined for classical hallmarks of senescence. COPD-derived fibroblasts showed elevated levels of senescence markers, γ-H2A.X-positive nuclei (indicative of DNA damage), and oxidative stress when compared to healthy controls. Importantly, these effects were most significant in the SEO-COPD subgroup. For example, SA-β-Gal positivity was significantly increased only in SEO-COPD fibroblasts, and elevated expression of p21^Cip1/Waf1^ in lung tissue was similarly confined to this group [46]. These findings align with genetic studies indicating that telomerase mutations are a risk factor for early-onset emphysema [117], supporting the hypothesis that intrinsic susceptibility to senescence may underlie more aggressive disease forms.

In contrast, studies exploring systemic senescence-inducing factors present a more nuanced picture. Kuźnar-Kamińska et al. investigated whether serum from COPD patients across GOLD stages 1–4 and risk groups A–D could induce a senescent phenotype in human bronchial epithelial cells (HBECs). Their findings revealed that COPD patient serum significantly increased the expression of senescence markers (SA-β-Gal, γ-H2A.X, p21^Cip1/Waf1^) in HBECs when compared to serum from healthy controls. However, no significant differences in senescence marker expression were observed between different GOLD stages or risk groups. Moreover, the levels of SASP components secreted by the exposed HBECs, including VEGF, CXCL8, and CXCL5 also showed no variation with disease severity [119]. These findings suggest a two-tiered model of senescence in COPD. Systemic senescence-promoting factors may be present early in disease and consistent across stages, constituting an underlying pro-senescent milieu. Localized accumulation of senescent cells within lung tissues, particularly in patients with severe or early-onset COPD may drive progressive tissue remodeling, inflammation, and functional decline. This model highlights the importance of distinguishing between senescence-inducing stimuli and the actual tissue burden of senescent cells. It further underscores the need for stratified therapeutic approaches that consider COPD phenotype, disease stage, and cell-type specific senescence profiles when designing senescence-targeted interventions.

### 4.2. Senescence in IPF

Senescent lung fibroblasts are increasingly recognized as a contributor to the pathogenesis of IPF, a progressive and irreversible interstitial lung disease marked by excessive ECM deposition and alveolar remodeling [142]. While telomere shortening and replicative stress have long been implicated in promoting fibroblast senescence in IPF, emerging evidence highlights the additional roles of oxidative stress, mitochondrial dysfunction, and chronic inflammation in driving this phenotype [143]. Multiple studies have investigated biomarkers of fibroblast senescence in IPF. Sanders and colleagues found that elevated plasma levels of Growth Differentiation Factor 15 (GDF15), IL-6, CRP, and TNFRII are associated with an increased risk of interstitial lung abnormalities (ILA), a precursor state to pulmonary fibrosis [144]. Primary lung fibroblasts derived from IPF patients exhibit increased expression of canonical senescence markers including SA-β-gal, p53, p16^Ink4a^, and p21^Cip1/Waf1^, alongside a robust SASP [95,145].

Recent studies have implicated STAT3 activation in driving early senescence and IPF progression, suggesting that modulation of STAT3 signaling could mitigate fibroblast dysfunction [142]. Senescent fibroblasts in IPF also exhibit increased expression of anti-apoptotic Bcl-2 family proteins (Bcl-W, Bcl-2, Bcl-XL) and decreased expression of pro-apoptotic factors (Bak, Bax), conferring resistance to cell death [146,147]. Additionally, age-associated upregulation of PAI-1 has been observed in murine lung fibroblasts. PAI-1 promotes fibrotic remodeling via a vitronectin-independent pathway mediated through its interaction with SorLA (sortilin-related receptor 1), which is upregulated in both mouse models and human IPF tissue [148,149]. Targeting the PAI-1–SorLA axis may thus represent a novel therapeutic approach in IPF.

At the tissue level, senescent IPF fibroblasts display expanded SASP profiles, including pro-inflammatory cytokines (IL-1β, IL-6, TNF-α), profibrotic growth factors (TGF-β, Connective Tissue Growth Factor (CTGF), Platelet-Derived Growth Factor (PDGF), chemokines (MCP-1, CXCL1), and matrix-degrading enzymes (MMP-2, MMP-9, MMP-12), which collectively foster a self-reinforcing fibrotic niche [95,150]. Notably, CTGF has been shown to induce fibroblast senescence via ROS accumulation and p53-mediated induction of p16^Ink4a^ [151]. Proteomic analyses further support a senescence-associated chromatin remodeling phenotype, including the accumulation of Nuclear Factor Kappa B (NF-κB) subunit p65 on chromatin in senescent fibroblasts [101].

Advances in single-cell transcriptomics have revealed substantial heterogeneity within the fibroblast compartment in IPF lungs. Studies by Tsukui et al. (2020) and Habermann et al. (2020) identified diverse fibroblast subtypes, including homeostatic fibroblasts, pathogenic myofibroblasts, and peribronchial fibroblasts [152,153]. However, it remains unclear which of these subpopulations are most prone to senescence and responsible for sustaining a chronic SASP. Integrating high-resolution single-cell RNA sequencing with spatial transcriptomics and senescence marker profiling may enable precise mapping of senescent fibroblast niches and clarify their interactions with neighboring senescent epithelial cells [152,154,155].

Additionally, applying RNA velocity and lineage tracing techniques may help determine whether activated fibroblast subtypes transition into a senescent state and whether this state is reversible under therapeutic pressure. These tools could illuminate the plasticity of fibroblast senescence in IPF and inform the development of subpopulation-specific senolytic therapies and targeted delivery systems. Taken together, senescent fibroblasts play a central role in shaping the profibrotic microenvironment of the IPF lung. Understanding the heterogeneity, plasticity, and molecular underpinnings of fibroblast senescence remains essential for designing precision interventions aimed at halting or reversing fibrotic progression (Figure 1). It is paramount to mention however that most of the existing literature focuses on studying the mechanism of senescence in lung fibroblasts in relation to IPF. But future work should focus on the crosstalk of senescent fibroblasts with neighboring cell milieu and the transitional cell states within a fibrotic versus a non-fibrotic tissue.

### 4.3. Lung Cancer

In the context of lung cancer particularly non-small cell lung cancer (NSCLC) and its predominant adenocarcinoma subtype cellular senescence exhibits a distinctly paradoxical or “dual” role. In early carcinogenesis, cellular senescence functions as a potent tumor-suppressive mechanism, interfering with the proliferation of cells with oncogenic alterations through stable/persistent cell cycle arrest and immune-mediated clearance. However, in advanced or established tumors, senescent cells—especially those that persist—can adopt a pro-tumorigenic phenotype via SASP. This chronic SASP output promotes inflammation, tumor progression, immune evasion, metastatic spread, and resistance to therapy [156]. For tumor progression, cancer cells must bypass this senescence barrier, frequently through the acquisition of loss-of-function mutations in p53 or p16^Ink4a^ [157].

OIS represents a critical tumor-suppressive barrier that acts to prevent malignant transformation at the earliest stages of carcinogenesis. In lung adenocarcinoma, activation of oncogenes such as KRAS or BRAF elicits a potent senescence response characterized by stable cell cycle arrest, effectively halting the proliferation of transformed cells [15]. This protective mechanism, often referred to as the “friend” role of senescence, helps to confine lesions to a pre-malignant or early malignant state. However, for tumor progression to occur, cancer cells must bypass this senescence barrier, frequently through the acquisition of loss-of-function mutations in tumor suppressors such as p53 or p16^Ink4a^ [157].

Paradoxically, once senescent cells persist particularly in the setting of TIS they often adopt a “foe” role in cancer progression. Senescent cells develop a robust SASP, which is a complex cocktail of pro-inflammatory cytokines (e.g., IL-6, IL-8), chemokines, growth factors, and proteases that remodel the tumor microenvironment (TME) [6]. This remodeling can promote epithelial–mesenchymal transition (EMT), angiogenesis, and invasion, thereby enhancing tumor aggressiveness [158,159].

Recent studies have demonstrated that senescent lung fibroblasts such as those found in IPF, a known risk factor for lung cancer, secrete exosomes enriched in matrix metalloproteinase-1 (MMP-1). These exosomes are readily internalized by NSCLC cells, where they activate the PI3K–AKT–mTOR pathway, stimulating cellular proliferation and clonogenicity [160]. The persistence of senescent cells following chemotherapy is therefore increasingly linked to adverse clinical outcomes, including tumor relapse and metastasis [161].

SASP also exerts profound effects on the immune landscape of the TME. While initially capable of recruiting immune cells for senescent cell clearance, a chronic SASP may shift the microenvironment toward immunosuppression. This phenomenon has been exemplified in KRAS-driven lung cancer models, where senescent macrophages have been identified as a major pro-tumorigenic population [162]. These macrophages, which are also found in aged lungs and pre-malignant human lung lesions, exhibit a unique SASP (specific to tumor cells) that promotes tumor progression. Notably, senolytic clearance of these cells using agents such as ABT-737 was shown to reduce tumor burden, enhance anti-tumor immunity, and extend survival in murine models [162].

These findings show the context-dependent duality of senescence in lung cancer. While senescence can initially restrain malignant transformation, its persistence especially in the TME facilitates tumor evolution and immune evasion. Critically, these effects are mediated by specific, targetable senescent cell populations, reinforcing the therapeutic potential of senolytics and SASP modulators in lung cancer treatment.

### 4.4. Senescence in Acute Lung InjuryALI and Acute Respiratory Distress Syndrome (ARDS) (Including COVID-19)

The role of cellular senescence in ALI and ARDS has emerged as a critical area of investigation, particularly in the wake of the COVID-19 pandemic. In contrast to chronic lung diseases where senescence is well established as a pathogenic driver the function of senescence in acute injury is more context-dependent and paradoxical. It often exhibits characteristics of a “double-edged sword”, exerting both protective and deleterious effects depending on the timing, duration, and cellular context of its activation [87,124,163].

In response to acute insults such as viral infection or severe inflammation, the lung initiates a transient senescence response that serves as an early protective mechanism [164]. This form of acute senescence acts to arrest the proliferation of damaged or infected cells, thereby limiting the spread of pathogens and preserving tissue integrity. Unlike apoptosis, this temporary growth arrest allows for cellular survival and immune-mediated clearance, preventing further tissue disruption [87].

A clear illustration of this process is observed in the context of severe COVID-19, where SARS-CoV-2 infection has been shown to induce a pronounced senescent phenotype in AT2 epithelial cells. These infected senescent cells exhibit elevated expression of key markers such as p16^Ink4a^ and the DNA damage marker γ-H2AX, indicating the activation of a canonical senescence program [126]. In the setting of ALI and LARDS (COPD, IPF, Asthma, etc.) the SASP plays a dual role in immunomodulation. Initially, the secretion of pro-inflammatory cytokines and chemokines facilitates a beneficial immune response, aiding in the recruitment of immune cells to eliminate pathogens, clear apoptotic or damaged cells, and remove senescent cells once their protective role is fulfilled [87]. This acute inflammatory signaling is prominently observed in severe COVID-19, where senescent AT2 epithelial cells exhibit markedly elevated expression of SASP cytokines such as IL-1β and IL-6. These factors contribute directly to the hyperinflammatory state that characterizes severe disease [87].

However, when senescent cells are not efficiently cleared, this initially protective SASP can become pathogenic. Persistent SASP signaling can promote chronic inflammation, impair tissue repair, and exacerbate lung damage. This failure of clearance is particularly pronounced in the context of immunosenescence, a state of age-associated immune dysfunction which limits the immune system’s capacity to resolve inflammation and remove senescent cells [87]. Thus, the context, duration, and immune competence of the host are critical determinants of whether the SASP functions as a beneficial or deleterious mediator in ALI and LARDS.

When the acute senescence program fails to resolve appropriately, it transitions from a protective mechanism into a pathogenic driver of chronic lung damage. This unresolved senescent state contributes to delayed resolution, fibrotic remodeling, and long-term impairment of pulmonary function [164]. The persistent secretion of SASP factors from an accumulating burden of senescent cells perpetuates a chronic inflammatory milieu, which affects with normal tissue regeneration and promotes the deposition of extracellular matrix, ultimately leading to fibrosis which can occur in long COVID-19 [87].

The accumulation of senescent alveolar epithelial cells significantly compromises the host’s capacity to recover, diminishing epithelial integrity and regenerative potential in ALI [165].

This chronic, unresolved senescent state characterized by persistent SASP signaling, immune dysfunction, and a loss of reparative capacity is increasingly recognized as a central mechanism underlying the post-acute sequelae observed in survivors of ARDS and severe COVID-19 [126]. These findings emphasize the need for therapeutic strategies aimed at modulating senescence resolution, restoring immune surveillance, and preserving epithelial regeneration in the aftermath of acute lung injury.

### 4.5. Cystic Fibrosis (CF)

Cellular senescence is a critical pathological feature in cystic fibrosis (CF), a genetic disease marked by chronic neutrophilic inflammation, mucus accumulation, and progressive lung tissue damage [166,167]. While CF was traditionally considered a pediatric disorder, advancements in treatment have significantly extended patient lifespan, uncovering hallmarks of premature lung aging in this population [166]. The CF airway epithelium exhibits a distinct senescence-associated signature, with elevated expression of canonical senescence markers including p16^Ink4a^, γH2A.X, and phospho-Chk2, when compared to non-CF controls. Notably, this senescent phenotype is spatially and cell-type-specific. Recent single-cell RNA sequencing revealed that basal and secretory epithelial cells within CF airways show the highest expression of senescence markers [168]. Intriguingly, even in the absence of inflammatory stimuli, CFTR-deficient bronchial epithelial cells exhibit a senescent-like phenotype characterized by elevated p21^Cip1/Waf1^ levels and reduced proliferative capacity, suggesting that CFTR dysfunction itself acts as a cell-intrinsic senescence. Among the extrinsic inducers of senescence in CF, neutrophil elastase (NE) is particularly important. NE, abundantly released into the CF airway due to sustained neutrophil infiltration, has been shown to induce cellular senescence in airway epithelial cells via p16^Ink4a^ mediated CDK4 inhibition [169,170]. This establishes a self-reinforcing loop, as senescent cells develop a SASP that exacerbates inflammation by recruiting additional immune cells, thus amplifying tissue damage and further promoting senescence [170,171]. Additionally, neutrophils isolated from bronchoalveolar lavage fluid of CF patients have been shown to express p21^Cip1/Waf1^, which may prolong their survival and contribute to persistent inflammation [167].

Emerging studies have begun to elucidate novel pathways that sustain senescence in CF beyond CFTR loss. The FGFR-MAPK-p38 axis has been implicated in promoting senescence in CF bronchial epithelium. Pharmacologic inhibition of FGFR4 significantly reduced senescence marker expression and improved mucociliary clearance in preclinical models, indicating this pathway’s therapeutic potential [168]. These findings suggest that although CFTR dysfunction initiates disease, secondary senescence may be maintained by CFTR-independent mechanisms, possibly explaining persistent inflammation in CF patients treated with highly effective modulator therapies [168]. Altogether, the accumulation of senescent cells in CF lungs not only accelerates structural damage but also contributes to chronic inflammation and impaired repair. As such, targeting cellular senescence represents a promising therapeutic avenue to mitigate disease progression, especially in the aging CF population [171].

### 4.6. Pulmonary Arterial Hypertension

Pulmonary Arterial hypertension (PAH) is a progressive and proliferative vascular disorder in which cellular senescence is increasingly recognized as a central pathological mechanism [172,173]. The accumulation of senescent cells, particularly pulmonary artery endothelial cells (PAECs) and pulmonary artery smooth muscle cells (PASMCs), contributes to the vascular remodeling that characterizes PH pathogenesis [174].

In both human idiopathic pulmonary hypertension, PH (Idiopathic Pulmonary Arterial Hypertension) and animal models, elevated expression of senescence markers such as p16^Ink4a^, p21^Cip1/Waf1^, and γH2AX has been consistently reported in vascular tissues [172,175]. Notably, hypoxia-induced senescence in PASMCs leads to paracrine secretion of IL-6, promoting the proliferation of neighboring PASMCs via the mTOR/S6K1 signaling axis [176].

In addition to paracrine signaling, juxtacrine interactions between senescent endothelial cells (ECs) and PASMCs have been shown to drive disease progression. Specifically, senescent ECs upregulate Notch ligands (e.g., Jagged-1 (JAG1), Delta-Like 4(DLL4), which activate Notch signaling in adjacent PASMCs. This mechanism was confirmed in EC-specific progeroid mouse models, where pharmacological inhibition of Notch signaling attenuated disease severity [177]. Transcriptomic analyses have further identified YWHAZ as a central hub gene in hypoxic PAECs and PH models; its silencing promotes autophagy, suggesting a potential strategy to facilitate senescent cell clearance [178,179]. Other pathways and molecules, such as SIRT1, mTOR, miRNA 21, epigenetic modifications, and mitophagy may be involved in cellular senescence of lung endothelial cells. This may also be associated with co-morbidity of IPF/COPD with PAH.

Senescence also appears to demarcate the irreversibility of PH progression. In a congenital heart disease-associated PH (PAH-CHD) rat model, the transition from reversible to irreversible pathology was marked by a shift toward a senescent vascular phenotype, with increased expression of p16^Ink4a^, p21^Cip1/Waf1^, MMP2, and IL-6. Remarkably, senolytic therapy with ABT263 reversed advanced PH in this model, supporting a causal role for senescent cell accumulation in disease progression [180]. This pro-fibrotic and pro-remodeling function of senescence is echoed in findings that frataxin deficiency, a known inducer of mitochondrial dysfunction, promotes endothelial senescence and PH, which is also amenable to senolytic intervention [173].

Paradoxically, recent data suggest that senescence may play a protective or homeostatic role in certain contexts. In contrast to the above findings, genetic (p16^Ink4a^-ATTAC model) and pharmacological (ABT263, Forkhead Box O4-Drug Resistance Inhibitor (FOXO4-DRI) clearance of senescent cells in various rodent PH models resulted in worsened hemodynamics and enhanced vascular remodeling [175]. These effects were attributed to the loss of senescent pulmonary endothelial cells (P-ECs), which represent a significant portion of the senescent population even in healthy lungs and may serve essential homeostatic and anti-proliferative functions [174,175].

### 4.7. Senescence in Asthma

Asthma, especially severe, late-onset, and Th2-low phenotypes, shows airway cellular senescence that intersects with chronic inflammation and remodeling. Repeated epithelial injury from allergens, viral exposures, pollutants, and oxidative stress drives DNA-damage responses, mitochondrial dysfunction, and checkpoint activation (p16/p21), producing a stable growth-arrested state with a pro-inflammatory, matrix-remodeling secretome that amplifies hyperresponsiveness and promotes fixed airflow limitation [111,122,181].

In the epithelium, thymic stromal lymphopoietin (TSLP) has been identified as an upstream trigger of senescence and remodeling. Human asthmatic airway biopsies display increased epithelial p16 and p21 expression together with collagen I and α-SMA, consistent with remodeling. Silencing p16 and p21 or pharmacologic STAT3 inhibition prevents the induction of collagen I and α-SMA in vitro. In allergen-challenged mice, STAT3 inhibition reduces epithelial senescence, diminishes airway remodeling, and alleviates hyperresponsiveness, supporting a causal role for TSLP-driven senescence in asthma pathology [111]. Airway smooth muscle (ASM) senescence is thought to participate by altering calcium handling, sustaining baseline contractile tone, and producing a SASP that stimulates fibroblast activation and ECM turnover. Crosstalk among senescent epithelial cells, ASM, and fibroblasts, enhanced by TSLP signaling, may reinforce hyperresponsiveness and steroid insensitivity [111].

Fibroblasts also display evidence of accelerated senescence. Bronchial fibroblasts isolated from mild asthmatic patients exhibit significantly shorter telomeres than those from healthy controls, with an average reduction from approximately 10.7 kb to 8.9 kb. They also show a markedly higher fraction of SA-β-gal-positive cells. Importantly, telomere length in fibroblasts correlates inversely with airway hyperresponsiveness, as measured by methacholine PC20, suggesting that replicative senescence contributes directly to disease severity [92].

Together, these data highlight biomarker patterns of airway senescence in asthma, including epithelial p16/p21 upregulation with loss of proliferative markers, epithelial and mesenchymal SA-β-gal activity, telomere attrition in bronchial fibroblasts, and induction of collagen I and α-SMA in remodeled tissue. These signatures are closely aligned with clinical features of severe asthma, including frequent exacerbations, poor bronchodilator response, and fixed obstruction, particularly in elderly-onset, obesity-associated, and T2-low endotypes [92,111].

From a therapeutic standpoint, senomorphics that dampen epithelial and mesenchymal senescence signaling, such as inhibitors of the TSLP–STAT3 axis or related inflammatory pathways, could reduce remodeling and restore treatment responsiveness. In preclinical models, STAT3 inhibition has been shown to attenuate both senescence and airway dysfunction. In parallel, airway-targeted senolytics may help selectively eliminate senescent cell populations that sustain remodeling, particularly if applied in short, pulsed regimens layered onto standard asthma therapies and biologics [92,111].

### 4.8. Key Proteins Implicated in Cellular Senescence in Lung Diseases

An array of proteins is critically involved in the complex process of cellular senescence in the lungs. These proteins participate in various pathways, including cell cycle regulation, DNA damage response, and the secretion of SASP factors, all of which contribute to lung aging and the pathogenesis of lung age-related diseases (Table 1).

#### 4.8.1. IL-10 (Interleukin 10)

Studies in mice with IL-10 (anti-inflammatory cytokine) gene knockouts have revealed that a deficiency in this protein exacerbates cellular senescence and accelerates lung fibrosis. These mice exhibit heightened inflammatory responses, and the introduction of recombinant IL-10 can mitigate these effects by reducing the expression of senescence markers p16 and p21. In vitro experiments have further demonstrated that the absence of IL-10 promotes the secretion of SASP factors from lung fibroblasts [199]. In the context of COPD, some studies have noted that mice with a deficiency in the IL-10 gene show an increase in senescent cells. Another report showed that IL-10 levels are reduced in the airways of patients with COPD [184,186].

#### 4.8.2. TP53 (p53)

The tumor suppressor protein p53 is a central figure in cellular senescence, primarily by governing cell cycle arrest and apoptosis in response to cellular stress. In conditions such as IPF and COPD, increased levels of p53 are observed in lung tissues. This upregulation of p53 is associated with the induction of apoptosis in alveolar epithelial cells, which can impair the regenerative capacity of the lung. Its role in cell cycle arrest is mediated through the transcriptional activation of Cyclin-Dependent Kinase Inhibitor 1A (CDKN1A; p21). In human lung fibroblasts, TP53 is also implicated in cell cycle arrest in the context of ARDS [35,83,86,94,131,182,183].

#### 4.8.3. H2AX (H2A.X Variant Histone)

H2A.X is a well-established marker of DNA double-strand breaks and is closely linked to cellular senescence. In aging and COPD, an increase in γ-H2AX in the lungs indicates an accumulation of DNA damage that can trigger cellular senescence [33,110]. Studies have shown that γ-H2AX accumulates at telomeres and in cells undergoing senescence. In IPF, a disease characterized by accelerated lung aging, increased levels of γ-H2AX are found in lung cells [200].

#### 4.8.4. Cyclin-Dependent Kinase Inhibitor 2A (CDKN2A; p16^INK4a^)

This gene encodes the p16^INK4a^ protein, a key inhibitor of cyclin-dependent kinases that plays a crucial role in inducing and maintaining cell cycle arrest in senescent cells. Knockout or silencing of CDKN2A in mouse models has been shown to affect cellular senescence in the lungs. The expression of p16^INK4a^ is considered one of the most specific markers of senescent cells and is frequently used to identify them in aging tissues [82,83,133,183,190,196,197].

#### 4.8.5. GDF15

It is a member of the TGF-β superfamily and is recognized as a component of the SASP. Its expression increases under cellular stress and in chronic lung diseases like COPD and IPF. In these diseases, elevated GDF15 levels serve as a biomarker and are associated with disease severity and progression [191,192,193,195].

#### 4.8.6. CDKN1A (p21)

p21 is a potent cyclin-dependent kinase inhibitor that is a downstream target of p53. Following cellular stress and DNA damage, p21 is induced and enforces cell cycle arrest, a hallmark of senescence. In lung fibrosis and ARDS, an increase in p21 expression is observed. While it can limit acute lung injury by preventing apoptosis, its sustained expression can also promote fibrosis by suppressing alveolar regeneration [29,33,35,83,86,131,183,188,189].

#### 4.8.7. TNFRSF1B (TNF Receptor Superfamily Member 1B)

TNFRSF1B shows increased expression in the lungs in hyperoxia-induced senescence [33]. This suggests a role for this receptor in inflammatory signaling pathways contributing to the SASP.

#### 4.8.8. Bcl2 L1 (BCL2 Like 1)

BCL2 L1, an anti-apoptotic protein, shows decreased expression in fibroblasts in the context of IPF [147].

#### 4.8.9. CXCL8 (IL-8)

CXCL8 is a component of the SASP whose expression is increased in senescent lung cells, particularly in IPF and COPD [184,185,201]. Produced by various cell types, CXCL8 is a chemoattractant for neutrophils, contributing to chronic inflammation and tissue damage in lung diseases.

#### 4.8.10. IL1A (Interleukin 1 Alpha)

IL1A is a pro-inflammatory cytokine that shows increased expression in senescent epithelial cells in the lungs, particularly in IPF [35,83,183,194]. Damaged epithelial cells release IL-1A, triggering an inflammatory response in neighboring fibroblasts, promoting fibrosis.

#### 4.8.11. MMP12 (Matrix Metallopeptidase 12)

MMP12 is a protease increasingly expressed in the lungs of IPF patients [35]. It contributes to tissue remodeling and fibrosis and is secreted by senescent alveolar epithelial cells. Its expression also increases after cigarette smoke exposure [83].

#### 4.8.12. SERPINE1 (PAI-1)

SERPINE1 is found at elevated levels in the lungs in contexts of cellular senescence, including IPF [35,94,198]. Increased SERPINE1 expression in AT2 cells can induce senescence via the p53-p21-Rb pathway [94].

#### 4.8.13. TGFβ1 (Transforming Growth Factor Beta 1)

TGFβ1 can induce senescence and is central to fibrosis. In mouse models, TGF-β1 signaling mediates senescence-associated pulmonary fibrosis [182]. In alveolar type-2 epithelial cells, TGFβ signaling is linked to a DNA damage response and senescence [183]. In IPF, the fibrogenic secretome of senescent cells highlights TGFB1’s role in disease progression [35].

#### 4.8.14. TNF (Tumor Necrosis Factor)

TNF is implicated in the SASP. In human and mouse models of IPF, increased senescence biomarkers are observed, and the secretome of senescent fibroblasts includes TNF, which is fibrogenic [35].

#### 4.8.15. IL-6 (Interleukin 6)

IL-6 is a prominent SASP factor. In COPD, senescent cells secrete elevated IL-6, contributing to chronic inflammation [184,185]. In IPF and cigarette smoke exposure models, IL-6 is also increased and is part of the fibrogenic secretome [35,83,194].

#### 4.8.16. IL-1βa (Interleukin 1 Beta)

IL-1β is a pro-inflammatory cytokine and SASP mediator. In COPD, increased IL-1β is found in the lungs and is associated with inflammatory processes [184].

#### 4.8.17. MMP-8 (Matrix Metallopeptidase 8)

MMP-8 is a protease involved in ECM degradation. In COPD, senescent cells secrete MMP8, contributing to tissue remodeling [187].

#### 4.8.18. VEGFA (Vascular Endothelial Growth Factor A)

VEGFA promotes angiogenesis and is part of the SASP in COPD. Increased VEGFA secretion by senescent cells contributes to pathological vascular remodeling [185].

#### 4.8.19. SIRT1

Anti-aging and anti-inflammatory protein which regulates cellular senescence, is downregulated in lungs of patients with COPD [137,138].

The above-mentioned markers (Table 1) are primarily inflammatory mediators or cell-cycle regulators; their functions are not specific to senescence but rather reflect downstream outcomes associated with the senescent state.

## 5. Therapeutics of Cellular Senescence

### 5.1. Senolytics

**Figure 3:**
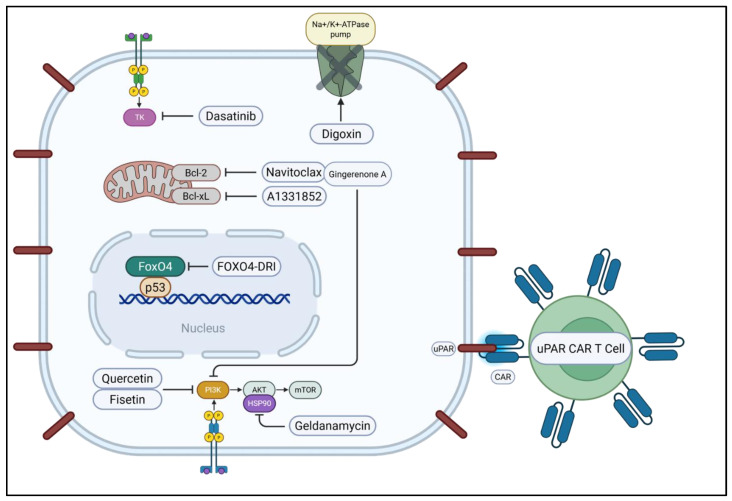
Mechanisms of action of senolytic drugs targeting pro-survival pathways in senescent cells. This schematic illustrates the primary intracellular mechanisms targeted by senolytic agents to selectively induce apoptosis in senescent cells. Senescent cells rely on distinct senescent cell anti-apoptotic pathways (SCAPs) for survival, including the Bcl-2 family, PI3K/AKT, tyrosine kinases, HSP90, and FOXO4-p53 interactions. Compounds such as navitoclax, fisetin, quercetin, and dasatinib disrupt these SCAPs, promoting mitochondrial-mediated cell death. Other agents like ouabain, digoxin, and CAR-T cells targeting uPAR eliminate senescent cells through plasma membrane or immune recognition mechanisms. Drug actions are depicted relative to their subcellular targets, with inhibitory effects shown using blunt arrows (⊣).

Senotherapeutics encompass two main strategies for targeting cellular senescence: senolysis and senomorphism. Senolytics are compounds designed to selectively induce apoptosis in SnCs, thereby reducing the burden of these cells in tissues [77,202]. This approach contrasts with senomorphics (or senostatics), which aim to suppress the deleterious SASP without necessarily eliminating the cells themselves [77,203]. The rationale behind senolysis is that the removal of SnCs can alleviate their detrimental contributions to aging and age-related diseases, potentially improving tissue function and extending healthspan [77,202] (Figure 3).

### 5.2. Mechanisms of Senolytic Action

The selective action of senolytics relies on exploiting the vulnerabilities inherent in the senescent state. To survive despite accumulating cellular damage and producing a potentially self-toxic SASP, SnCs upregulate a network of pro-survival pathways, collectively termed senescent cell anti-apoptotic pathways (SCAPs) [77,204,205]. These pathways confer resistance to apoptosis. Senolytics function by transiently disabling these specific SCAPs, rendering SnCs susceptible to their pro-apoptotic microenvironment or internal damage signals [77,202].

Bioinformatic analyses of senescent versus non-senescent cells identified several key SCAPs, including pathways involving Ephrins, PI3K/AKT, p53/p21^Cip1/Waf1^/Serpins, HIF-1α, and notably, the BCL-2 family of anti-apoptotic proteins [77,202,204]. The dependency on specific SCAPs can vary between different types of SnCs, highlighting the heterogeneity of senescence and suggesting why certain senolytics exhibit cell-type specificity [77,204].

### 5.3. Major Classes and Examples of Senolytic Agents

Research into senolytics has identified promising candidates from diverse sources, including natural products, repurposed drugs originally developed for other indications, and novel synthetic molecules specifically designed to target senescence pathways (Table 2).

### 5.4. Early Senolytics (Dasatinib and Quercetin)

The first senolytics reported, dasatinib (D) and quercetin (Q), were identified through a hypothesis-driven, bioinformatics-based approach targeting SCAPs [205]. Dasatinib is a tyrosine kinase inhibitor used clinically for leukemia, while quercetin is a natural flavonoid known to interact with PI3K and Bcl-2 family members [77,202,206]. While each compound has senolytic activity against specific cell types (e.g., dasatinib against senescent preadipocytes, quercetin against senescent HUVECs), their combination (D+Q) exhibits broader efficacy, targeting a wider range of SnCs and often showing synergistic effects [77,202]. Preclinical studies demonstrated that D+Q administration can alleviate numerous age-related dysfunctions in mice, including improving cardiovascular function, reducing osteoporosis, mitigating frailty, and improving physical function even when administered late in life [202]. Furthermore, D+Q treatment has shown benefits in models of atherosclerosis, pulmonary fibrosis, hepatic steatosis, neurodegeneration, and metabolic dysfunction associated with diabetes or obesity [77,108,202]. D+Q treatment also protected against cisplatin-induced ovarian injury by removing SnCs [109].

### 5.5. Natural Products

#### 5.5.1. Fisetin

A flavonoid structurally related to quercetin, found in fruits and vegetables, has shown potent senolytic activity [77,207]. It targets multiple pathways including PI3K/AKT and Bcl-2 family members [207]. Fisetin selectively induces apoptosis in senescent HUVECs and certain fibroblasts but not primary human preadipocytes [77,207]. In vivo studies reported that fisetin extends health and lifespan in mice [208].

#### 5.5.2. Gingerenone A

A component isolated from ginger extract, was recently identified as a novel senolytics [209]. It selectively eliminated senescent human fibroblasts (WI-38) without significant effect on proliferating cells. Its mechanism involves inducing apoptosis via caspase-3 activation and reducing the anti-apoptotic protein Bcl-xL, acting independently of p53. Gingerenone A also exhibited senomorphic properties by suppressing aspects of the SASP, including IL-6 secretion [209].

Other natural products like piperlongumine and curcumin analogues (EF-24) have also been reported as senolytics [77,202,210].

#### 5.5.3. Bcl-2 Family Inhibitors

Given the role of anti-apoptotic Bcl-2 family proteins (Bcl-2, Bcl-xL, Bcl-w) in SnC survival, inhibitors of these proteins have been investigated as senolytics.

#### 5.5.4. Navitoclax (ABT-263)

An orally bioavailable inhibitor of Bcl-2, Bcl-xL, and Bcl-w, demonstrated senolytic activity in multiple cell types (senescent HUVECs, IMR90 fibroblasts, MEFs) but notably not in primary human preadipocytes [204]. Navitoclax treatment reduced SnC burden and rejuvenated aged hematopoietic stem cells in mice [211]. However, its clinical translation for senolysis is hampered by significant dose-limiting toxicities, particularly thrombocytopenia and neutropenia, likely due to its inhibition of Bcl-xL in platelets and Bcl-2 in neutrophils [77,204].

#### 5.5.5. Selective Bcl-xL Inhibitors

Agents like A1331852 and A1155463, which primarily target Bcl-xL, also exhibit senolytic activity in HUVECs and IMR90 cells, but not preadipocytes [77,202,207]. By sparing Bcl-2, they are hypothesized to have reduced hematological toxicity compared to navitoclax, although this requires further investigation [77].

#### 5.5.6. HSP90 Inhibitors

Heat shock protein 90 (HSP90) is a crucial chaperone for stabilizing numerous client proteins involved in cell survival and proliferation, including AKT [212]. Several HSP90 inhibitors, such as 17-DMAG (alvespimycin), geldanamycin, and 17-AAG (tanespimycin), originally developed as anti-cancer agents, were identified as a novel class of senolytics [202,213]. These compounds induce apoptosis in various types of senescent cells (e.g., MEFs, human fibroblasts, HUVECs). Mechanistically, they disrupt the HSP90-AKT interaction, leading to the destabilization and degradation of active AKT [212,213]. In vivo, intermittent treatment with 17-DMAG reduced tissue senescence markers and extended health span in the Ercc1^−/Δ^ progeroid mouse model [213].

### 5.6. Additional Classes of Senolytics

Additional classes with senolytic activity include cardiac glycosides (e.g., ouabain, digoxin) which inhibit Na^+^/K^+^-ATPase [214], and compounds targeting the p53 pathway, such as FOXO4-DRI peptides which disrupt the FOXO4-p53 interaction [215], and inhibitors of MDM2 or USP7 which stabilize p53 [77]. Aspirin has also been reported to have senolytic properties in certain contexts [202].

Beyond the known classes of senolytics such as tyrosine kinase inhibitors (Dasatinib), flavonoids (Quercetin, Fisetin), and Bcl-2 family inhibitors (Navitoclax), several novel strategies have recently emerged to improve selectivity and efficacy while minimizing off-target effects. For instance, β-galactosidase-activated prodrugs such as Galacto-Navitoclax exploit the elevated SA-β-Gal activity in senescent cells to release cytotoxic payloads selectively, demonstrating enhanced precision in preclinical models [216]. Similarly, PROTAC-based senolytics like ARV825 have been designed to degrade anti-apoptotic or epigenetic survival proteins specifically in senescent cell populations, offering a promising next-generation approach [217,218].

Additionally, MDM2 inhibitors such as Nutlin-3a can reactivate p53 pathways to induce apoptosis in senescent or stressed cells [211]. Selective targeting of other anti-apoptotic proteins is also gaining traction; for example, Mcl-1 inhibitors (e.g., S63845) have shown potential for removing resistant senescent cells, though primarily explored in oncology thus far [219]. Finally, the development of nanoparticle-encapsulated senolytics, including lung-targeted formulations of Quercetin or Dasatinib, represents an innovative strategy to localize senolytic activity, potentially reducing systemic toxicity and improving therapeutic index for lung diseases [220].

### 5.7. Novel Therapeutic Modalities

Beyond small molecules, innovative approaches are being developed.

#### Senolytic CAR-T Cells

Chimeric antigen receptor (CAR) T cell technology has been adapted for senolysis. By redirecting T cells to target proteins upregulated on the surface of SnCs, such as urokinase plasminogen activator receptor (uPAR), senolytic CAR T cells can effectively eliminate SnCs [217,218]. In preclinical models of physiological aging and diet-induced obesity, a single administration of anti-uPAR CAR T cells led to long-lasting clearance of uPAR-positive senescent cells, improved metabolic function (including glucose tolerance and insulin sensitivity), and enhanced physical capacity (Figure 3). Notably, these CAR T cells persisted for extended periods and demonstrated prophylactic effects, preventing metabolic decline when administered early in life [214]. This approach highlights the potential for durable effects from cell-based senolytic therapies.

### 5.8. Senomorphics

While the elimination of SnCs via senolytics has garnered significant attention, an alternative and potentially complementary therapeutic strategy involves modulating the characteristics of SnCs without inducing cell death (Table 3). This approach utilizes agents termed as senomorphics or senostatics. The core principle behind senomorphism is the suppression or alteration of the detrimental aspects of the SASP [1,77,103].

**Figure 4:**
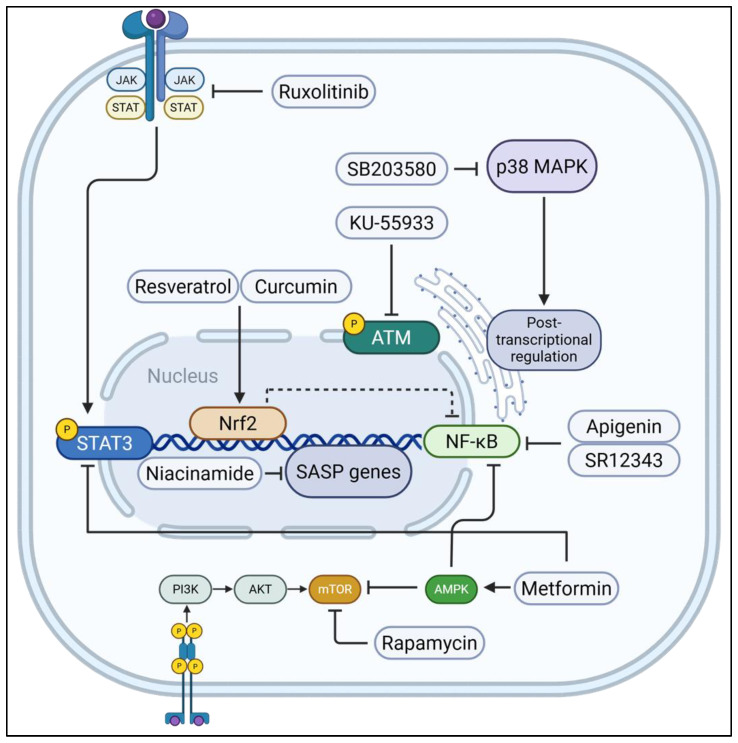
Mechanisms of action of senomorphic drugs targeting inflammatory and stress-related pathways in senescent cells. This schematic illustrates key intracellular pathways modulated by senomorphic agents to suppress the SASP. Stress signals activate the p38 MAPK–MK2 axis in the cytoplasm, which stabilizes SASP mRNAs and promotes inflammatory cytokine expression. Compounds such as SB203580, UR13756, and BIRB796 inhibit p38 MAPK to prevent downstream activation of MK2 and SASP production at the post-transcriptional level. Other senomorphic agents (e.g., rapamycin, metformin, resveratrol, curcumin) act by modulating mTOR, AMPK, NF-κB, STAT3, or Nrf2 pathways, affecting SASP at transcriptional or regulatory levels. Drug targets are shown relative to their subcellular localization, and inhibitory actions are indicated by blunt arrows (⊣).

The rationale for developing senomorphics stems from several considerations. Firstly, the SASP itself is recognized as a major driver of the deleterious effects associated with SnC accumulation [1,77]. Targeting the SASP directly offers a way to mitigate these effects. Secondly, SnCs are not uniformly detrimental; they play essential roles in physiological processes such as tumor suppression, embryonic development, and wound healing [8,77]. Senolytic therapies risk ablating these beneficial functions by eliminating SnCs. Senomorphics, on the other hand, might offer a more nuanced intervention by preserving the SnCs while neutralizing their harmful secretion. Thirdly, the heterogeneity of SnCs and their SASP profiles across different tissues, cell types, and inducing stimuli [7,77] presents a challenge for broadly effective senolytics. Senomorphic agents targeting common SASP regulatory pathways might provide a more universally applicable approach. By altering the SnC phenotype rather than inducing cell death, senomorphics aim to achieve a state of “senostasis,” reducing the pro-aging impact of senescent cells [202] (Figure 4).

### 5.9. Mechanisms of Senomorphic Action: Targeting SASP Regulation

Senomorphic compounds exert their effects primarily by interfering with the signaling pathways and molecular machinery responsible for producing and secreting SASP components. The SASP is remarkably complex, comprising hundreds of distinct factors including pro-inflammatory cytokines (e.g., IL-6, IL-8), chemokines (e.g., MCP-1/CCL2), growth factors, proteases (e.g., matrix metalloproteinases-MMPs), bioactive lipids, extracellular vesicles (EVs), and other molecules [7,63]. The production of this diverse secretome is tightly regulated at multiple levels, transcriptional, post-transcriptional, and translational, providing various points for senomorphic intervention [77]. Among various regulatory mechanisms, following pathways stand out as the principal drivers of inflammatory SASP factors:

#### 5.9.1. NF-κB Pathway

The nuclear factor-κB (NF-κB) pathway is a master regulator of inflammation and immunity and is critically involved in the transcription of numerous SASP factors, particularly pro-inflammatory cytokines like IL-6 and IL-8 [77,101]. Persistent DDR signaling, often present in SnCs, can lead to chronic NF-κB activation. Senomorphics targeting NF-κB signaling, either by inhibiting upstream kinases like IKK or preventing NF-κB nuclear translocation, can effectively dampen a significant portion of the inflammatory SASP [77].

#### 5.9.2. mTOR Pathway

The mammalian target of rapamycin (mTOR) pathway, particularly mTOR complex 1 (mTORC1) regulates cell growth, proliferation, and metabolism. It also plays a significant role in SASP regulation, primarily at the translational level. mTORC1 promotes the translation of specific mRNAs encoding SASP components, including the upstream SASP regulator IL-1α [77]. Inhibitors of mTOR, such as rapamycin, are potent senomorphics that suppress SASP production [77,202].

#### 5.9.3. p38 MAPK Pathway

The stress-activated p38 mitogen-activated protein kinase (MAPK) pathway is triggered by various cellular stresses, including those that induce senescence. p38 MAPK contributes to both senescence entry and SASP regulation, partly through stabilizing mRNAs of SASP factors and potentially converging with NF-κB activation [61]. Inhibitors of p38 MAPK have demonstrated senomorphic activity by reducing SASP secretion [77].

#### 5.9.4. JAK/STAT Pathway

The Janus kinase/signal transducer and activator of transcription (JAK/STAT) pathway is implicated in regulating specific SASP components, particularly those involved in immunosuppression [63]. JAK inhibitors, such as ruxolitinib, have shown promise as senomorphics by suppressing SASP factors and alleviating frailty in preclinical models [221].

#### 5.9.5. ATM Pathway

Ataxia telangiectasia mutated (ATM), a key kinase in the DDR pathway, is persistently activated in many SnCs. Beyond its role in cell cycle arrest, ATM contributes to SASP regulation, potentially through modulating NF-κB activity [80]. ATM inhibitors have shown senomorphic potential by reducing SASP expression in vitro and in vivo [77].

By targeting these central regulatory nodes, senomorphics can potentially normalize the microenvironment perturbed by SnCs, reduce chronic inflammation, and restore aspects of tissue homeostasis (Figure 4).

### 5.10. Natural Compounds and Derivatives

#### 5.10.1. Rapamycin (Sirolimus)

Isolated from *Streptomyces hygroscopicus* found on Easter Island, rapamycin is the archetypal senomorphic [202]. Its mechanism involves inhibiting mTORC1 signaling [222]. Extensive preclinical studies have demonstrated its ability to suppress SASP components, reduce cellular senescence markers, ameliorate age-related dysfunction in various models, and extend lifespan in species ranging from yeast to mice [77,202]. Despite its promise, clinical use for broad anti-aging purposes is hampered by potential side effects including metabolic dysregulation and immunosuppression, possibly linked to off-target inhibition of mTORC2 [77]. Development of rapalogs with improved specificity and pharmacokinetic profiles is ongoing [77].

#### 5.10.2. Resveratrol

This polyphenol, found in grapes and other plants, activates SIRT1 (though SRT1720 is a pharmacological activator [137]), an NAD^+^-dependent deacetylase involved in regulating metabolism, stress resistance, and aging [77]. Resveratrol exhibits complex, dose-dependent effects. At lower concentrations, it often acts as a senomorphic, suppressing SASP factors (e.g., by inhibiting NF-κB and activating Nrf2 pathways) and preventing senescence induction [77]. At higher concentrations, it can act as a pro-oxidant and induce senescence or apoptosis [77]. Its efficacy in extending lifespan in mice appears context-dependent (e.g., beneficial on high-fat diets but not standard diets), and its poor bioavailability remains a challenge [77]. Sirtuin-activating compounds (STACs) with potentially improved properties are under development [77].

#### 5.10.3. Curcumin

The primary bioactive compound in turmeric, curcumin exhibits broad biological activities. It has demonstrated senomorphic potential by down-regulating Nrf2 and NF-κB pathways involved in SASP production [77]. However, like resveratrol, curcumin suffers from poor bioavailability, limiting its systemic efficacy. Its analogue EF-24, while having improved bioavailability, is primarily characterized as a senolytic targeting BCL-2 family proteins [77].

#### 5.10.4. Other Flavonoids (Apigenin, Kaempferol, Quercetin)

Apigenin and kaempferol have shown senomorphic activity, potentially via inhibiting IRAK1/IκBα/NF-κB signaling [77]. Quercetin, while predominantly known as a senolytic, also exhibits senostatic properties in certain contexts, such as suppressing pro-inflammatory responses when delivered via functionalized nanoparticles [77,203]. This highlights the potential dual roles of some compounds.

#### 5.10.5. Niacinamide (Vitamin B3) and Hyaluronic Acid

A recent study demonstrated that a topical formulation combining 6% niacinamide and hyaluronic acid fragments exerted senomorphic effects in human skin biopsies taken after two months of clinical application [223]. This was evidenced by significant downregulation of SASP-related genes, including the DAMPs S100A8 and S100A9, MMP12, and the chemokine CXCL9. These molecular changes correlated positively with observed clinical improvements in skin radiance, smoothness, and homogeneity, providing a direct link between senomorphic action and cosmetic benefit in skin aging [223].

### 5.11. Repurposed Drugs

#### 5.11.1. Metformin

This biguanide drug, a cornerstone of type 2 diabetes treatment, is perhaps the most extensively studied repurposed drug for geroprotection [77,202]. Its senomorphic effects are thought to be mediated through multiple pathways, including AMPK activation, inhibition of NF-κB signaling, and modulation of STAT3, DICER1, and Nrf2/GPx7 activity [224]. It reduces SASP factors in various cell types and improves health span in model organisms [77,202]. The ongoing Targeting Aging with Metformin (TAME) trial is specifically designed to test its efficacy in targeting fundamental aging processes in humans [77].

#### 5.11.2. Statins

Primarily used for lowering cholesterol, certain statins (atorvastatin, pravastatin, pitavastatin, simvastatin) have shown potential to inhibit oxidative stress-induced endothelial senescence and suppress SASP factors [77]. Proposed mechanisms include Akt activation leading to eNOS/SIRT1 upregulation, and inhibition of protein prenylation affecting Rho GTPase activity, which can modulate actin dynamics and potentially SASP secretion [77]. However, potential side effects like muscle problems and increased diabetes risk need consideration [77].

#### 5.11.3. Aspirin

This common non-steroidal anti-inflammatory drug has shown complex, context-dependent effects on senescence. While some studies suggest it can inhibit senescence and SASP in endothelial cells, others indicate it can induce senescence in colorectal cancer cells [77]. Its senomorphic potential requires further investigation to clarify dose- and cell-type-specific effects.

#### 5.11.4. JAK Inhibitors (Ruxolitinib)

Given the role of JAK/STAT signaling in mediating responses to SASP cytokines and potentially regulating immunosuppressive SASP components, JAK inhibitors like ruxolitinib have been tested as senomorphics. Ruxolitinib suppressed SASP production and alleviated frailty in aged mice [77], supporting this pathway as a viable senomorphic target.

#### 5.11.5. ATM Inhibitors (KU-55933, KU-60019)

Since persistent ATM activity contributes to senescence and SASP, its inhibition has been explored. ATM inhibitors reduced NF-κB activation and SASP expression, alleviating senescence phenotypes in vitro and in Ercc1−/Δ progeroid mice [77]. However, concerns about potentially increasing cancer risk due to inhibiting a key DNA repair kinase necessitate careful evaluation [77].

#### 5.11.6. p38 MAPK Inhibitors (SB203580, UR13756, BIRB796)

Targeting the p38 MAPK pathway, which is activated by senescence-inducing stress and contributes to SASP regulation, is another senomorphic strategy. Inhibitors of p38 MAPK or its downstream effector MK2 effectively suppressed SASP secretion in various SnC models [77].

### 5.12. Novel Synthetic Compounds

#### NF-κB Inhibitors (SR12343)

Directly targeting the central SASP regulator NF-κB is a rational approach. The small molecule SR12343, developed as a specific IKK/NF-κB inhibitor, successfully reduced senescence and SASP in vitro and improved tissue pathologies and healthspan in aged and progeroid mouse models, demonstrating the potential of synthetic senomorphics [77].

Research is actively pursuing other synthetic molecules targeting SASP regulatory pathways or specific SASP components, aiming for greater potency, selectivity, and improved drug-like properties compared to existing agents.

### 5.13. Therapeutic Potential and Limitations of Senomorphism

Senomorphics offer a distinct therapeutic paradigm for tackling aging and age-related diseases. By focusing on neutralizing the harmful environment created by SnCs rather than eliminating the cells themselves, they may provide a safer long-term strategy, particularly considering the potential beneficial roles of SnCs and the risks associated with broad senolysis [77,202]. This approach could be particularly valuable for chronic conditions where sustained suppression of inflammation and tissue protection is desired. Evidence from preclinical models using agents like rapamycin, metformin, and specific pathway inhibitors (NF-κB, JAK, ATM) suggests that senomorphic interventions can indeed improve health span and alleviate symptoms associated with aging and specific diseases [77]. The recent demonstration of clinical benefit from a topical senomorphic formulation in skin aging further supports this potential [223].

Despite this promise, the development and application of senomorphics face significant hurdles [77]. A primary challenge lies in the complexity and context-dependency of the SASP [7]. Indiscriminate suppression of all SASP factors may be detrimental, as some components might have protective or necessary physiological functions (e.g., immune recruitment, tissue repair signals). Achieving selective modulation of only the pathogenic SASP components requires a much deeper understanding of SASP composition and regulation in different physiological and pathological contexts. Long-term safety is a major concern, especially for agents targeting fundamental pathways like mTOR, NF-κB, or ATM, which have vital roles beyond SASP regulation. Chronic inhibition of SASP could lead to unforeseen adverse effects, including impaired immunity, metabolic disturbances, or potentially increased cancer risk [77]. Developing robust biomarkers to specifically monitor senomorphic activity (i.e., effective SASP suppression) in vivo and in clinical trials is crucial for assessing efficacy and guiding dosing regimens, yet such biomarkers are currently lacking. Furthermore, optimizing drug delivery to ensure adequate and sustained levels of senomorphic agents in target tissues while minimizing systemic exposure remains a significant pharmacokinetic challenge [77]. Finally, the heterogeneity of senescence itself means that the effectiveness of a given senomorphic might vary considerably depending on the specific type of SnCs present and the underlying cause of their senescence. Overcoming these limitations will require continued basic research into SASP biology, careful preclinical safety and efficacy testing, innovative drug design and delivery strategies, and thoughtfully designed clinical trials (Table 4).

Together, these emerging modalities expand the senotherapeutic arsenal and underscore the need for disease-specific delivery systems and rigorous preclinical validation, particularly in fibrotic lung diseases such as IPF.

### 5.14. Delivery Mechanisms for Senotherapeutics

Effective and targeted delivery remains a challenge for many senotherapeutics, particularly natural products with poor bioavailability or agents with potential off-target toxicities. Nanotechnology offers promising solutions.

#### 5.14.1. Nanoparticles

Various nanoparticle formulations are being explored to improve senolytic delivery, stability, and targeting [203,206]. For example, quercetin surface-functionalized magnetic iron oxide nanoparticles (MNPQ) exhibited both senolytic and senostatic activity in cultured human fibroblasts undergoing oxidative stress-induced senescence, mediated partly through AMPK activation [203]. Nanoparticles can be engineered with specific ligands or properties to enhance accumulation in target tissues or uptake by SnCs [202,206].

#### 5.14.2. Exosomes

These naturally occurring EVs can act as delivery vehicles. Engineering exosomes to carry senolytic drugs represents another strategy for targeted therapy, although primarily explored in cancer contexts thus far [225].

#### 5.14.3. Galactose-Modified Prodrugs

This approach leverages the increased SA-β-gal activity found in the lysosomes of many SnCs. Cytotoxic drugs are conjugated to a galactose moiety, rendering them inactive. Upon uptake by SnCs, the elevated SA-β-gal activity cleaves the galactose, releasing the active drug selectively within the senescent cell [77,202]. Examples include Nav-Gal (a navitoclax prodrug) and SSK1 (a gemcitabine prodrug), which demonstrated enhanced specificity and reduced toxicity compared to the parent compounds in preclinical models [226].

#### 5.14.4. Antibody-Drug Conjugates (ADCs)

Building on recent findings by Poblocka et al. (2021), ADCs that target senescent cell surface markers, such as β_2_-microglobulin, offer a promising strategy for treating lung diseases [227]. The study showed that selective clearance of senescent cells using a B2M-directed ADC effectively reduced SASP-mediated inflammation and tissue remodeling. This precision-targeted approach holds therapeutic potential for managing chronic lung conditions, such as IPF and COPD, while minimizing off-target effects. Such strategies may ultimately help restore lung homeostasis and counter age-related pulmonary decline [227].

### 5.15. Therapeutic Applications and Strategies

The broad involvement of SnCs in numerous pathologies suggests that senolytics could have wide-ranging therapeutic applications, including for osteoarthritis, neurodegenerative diseases (e.g., Alzheimer’s disease), metabolic disorders (e.g., type 2 diabetes, obesity-related dysfunction), cardiovascular diseases, IPF, and frailty [77,103,108,202,214].

Given the heterogeneity of SnCs and their SCAPs, combination strategies are being considered. Combining senolytics with different mechanisms or targets (e.g., D+Q) may broaden the spectrum of SnCs eliminated and enhance overall efficacy [77,202]. Additionally, combining senolytics with senomorphics could offer a dual approach, reducing SnC burden while also suppressing the SASP from remaining or newly formed SnCs [77].

A key strategic advantage proposed for senolytics is the potential for intermittent dosing. Since SnCs accumulate relatively slowly, periodic administration (“hit-and-run” approach) might be sufficient to maintain a low SnC burden and achieve therapeutic benefits, while minimizing the potential for side effects associated with continuous drug exposure [108,202]. The long-lasting effects observed with senolytic CAR T cells further support the feasibility of intermittent or even single-dose curative therapies [214].

Despite significant progress, the development of senolytics faces challenges. The heterogeneity of SnCs necessitates careful selection of senolytic agents based on the specific cell types involved in a particular disease [77,202]. Off-target toxicity remains a concern, as exemplified by navitoclax, underscoring the need for highly selective agents or targeted delivery systems [204]. Furthermore, long-term safety data, particularly concerning potential effects on tissue repair, immune function, and tumorigenesis, are still needed [103,202]. Ongoing research focusing on identifying more specific senolytic targets, developing novel delivery platforms like PROTACs and advanced nanocarriers, refining immunotherapy approaches, and conducting well-designed clinical trials will be crucial for realizing the full therapeutic potential of senolytics in combating age-related diseases [77,202,214].

#### 5.15.1. Current Status of Clinical Trials

The field of senotherapeutics has witnessed rapid translation from preclinical discoveries to initial human clinical trials, representing a significant step towards targeting fundamental aging processes for the treatment of age-related diseases [77,228]. While still in early stages, clinical investigations are exploring the potential of senolytic agents, which selectively eliminate SnCs, and senomorphic agents, which modulate the SASP, across a spectrum of conditions [77,202].

Several senolytic agents, primarily repurposed drugs or natural compounds, are currently under investigation in human clinical trials [77,228]. The combination of the tyrosine kinase inhibitor dasatinib (D) and the flavonoid quercetin (Q) was among the first senolytic strategies to enter clinical testing [77,228]. Early phase trials have yielded encouraging, albeit preliminary, results. The first-in-human, open-label pilot study investigated intermittent D+Q administration in patients with IPF [77,228]. This study reported improvements in physical function, such as gait speed and chair-rise times, although direct evidence of SnC clearance in the lung was not assessed [127,228]. Another phase 1 trial evaluated D+Q in patients with diabetic kidney disease (DKD). Findings indicated that a short course of D+Q reduced the burden of senescent cells, measured by markers like p16^Ink4a^, p21^Cip1/Waf1^, and SA-β-gal activity, in subcutaneous adipose tissue biopsies collected 11 days after treatment cessation [228]. This study also observed a reduction in circulating SASP factors [228]. Ongoing trials are further evaluating D+Q in conditions including Alzheimer’s disease, chronic kidney disease, frailty in hematopoietic stem cell transplant survivors, and skeletal health [77,228].

Fisetin, another naturally occurring flavonoid related to quercetin, has also entered clinical investigation based on promising preclinical data demonstrating its senotherapeutic potential and ability to extend healthspan and lifespan in mice [208]. Current trials are assessing fisetin’s efficacy in conditions including frailty, chronic kidney disease, osteoarthritis, and COVID-19 [228]. Additionally, trials involving adult survivors of childhood cancer are planned [228]. Results from these trials are largely pending but anticipated with significant interest.

Other senolytic agents, such as BCL-2 family inhibitors (for example, navitoclax (ABT-263)), have also been identified and show promise in preclinical settings [77,204], although their clinical development specifically for senescence-related indications is less advanced compared to D+Q and fisetin, partly due to concerns about side effects like thrombocytopenia [229]. The development pipeline includes diverse agents targeting various SCAPs [77,202] (Table 4).

#### 5.15.2. Senotherapeutics Challenges and Opportunities

Despite the promising advances, the clinical development of senotherapeutics faces significant challenges [77,202]. One major hurdle is the delivery and targeting of senotherapeutics. Achieving selective elimination of SnCs while minimizing damage to healthy, non-senescent cells is critical for safety and efficacy [103]. The heterogeneity of SnCs across different tissues, developmental stages, and disease contexts further complicates targeted delivery. Innovations in drug delivery systems offer potential solutions. Nanotechnology, including the use of nanoparticles and exosomes, is being explored to enhance the targeted delivery of senotherapeutics [203,206,225]. For instance, quercetin functionalized onto Fe_3_O_4_ nanoparticles has shown senolytic activity in vitro [203], and engineered exosomes have been investigated as natural drug delivery vehicles for cancer therapy, a concept potentially adaptable to senotherapeutics [225]. Furthermore, galactose-modified prodrugs, designed to be activated specifically by the high β-galactosidase activity characteristic of many SnCs, represent another promising strategy to improve target specificity [230].

Safety considerations are paramount, particularly as senotherapeutics may eventually be considered for chronic or preventive use in aging populations [103,228]. Potential off-target effects remain a concern, as senolytics often target pathways crucial for the survival of other cell types, exemplified by the thrombocytopenia associated with certain BCL-2 inhibitors like navitoclax [77,229]. Modulation of the immune system, either directly by the drug or indirectly via alteration of the SASP or removal of SnCs that interact with immune cells, requires careful evaluation [103]. Long-term safety data are currently lacking, as clinical trials have involved relatively short-term interventions [228]. Robust biomarkers are needed not only to demonstrate target engagement (SnC clearance) but also to monitor potential adverse effects during clinical development [7].

#### 5.15.3. Biomarker Gaps: Need for In Vivo Markers of Senescence in Lungs

Despite increasing interest in targeting senescence for the treatment of chronic lung diseases, a major translational barrier lies in the absence of robust, specific, and non-invasive in vivo biomarkers capable of reliably detecting senescent cells within lung tissue. Current identification strategies predominantly rely on ex vivo or histological markers such as p16^Ink4a^, p21^Cip1/Waf1^, SA-β-gal activity, and DNA damage indicators like γH2AX [1]. However, these markers are hindered by limited specificity, restricted tissue accessibility, and an inability to differentiate between transient (e.g., pseudosenescent (Table 5) and truly senescent states, especially in complex tissues such as lung.

Efforts to develop in vivo imaging tools, including PET tracers that detect SA-β-gal activity (e.g., Galacto-rhodamine-based probes), are underway but currently lack validation in lung-specific disease models [231]. Meanwhile, transcriptomic-based senescence classifiers, such as SenMayo and SeneScore, have shown promise in characterizing senescent cell populations using bulk and single-cell RNA-sequencing data across tissues [7,232,233,234,235]. Nevertheless, these molecular signatures remain largely experimental and not yet adaptable for clinical use, particularly in the lung.

Emerging fluid-based biomarkers in bronchoalveolar lavage fluid (BALF), sputum, and serum such as SASP components and circulating mitochondrial DNA (mtDNA) released by senescent cells represent a promising non-invasive approach. However, these candidates remain insufficiently validated for lung-specific applications, and their capacity to distinguish between deleterious versus reparative senescence is unclear.

Altogether, these limitations underscore an urgent need for the development of lung-specific, senescence-selective biomarkers that can: discriminate senescent from non-senescent cells in vivo, and differentiate pathologic from physiologic senescence, and serve as pharmacodynamic markers for therapeutic response to senolytic or senomorphic agents.

## 6. Cellular Senescence in Lung Cells and Lung Diseases: Underexplored Aspects

Building upon these foundational insights, several emerging areas have garnered increasing attention, underscoring the need for further investigation to comprehensively delineate the multifaceted role of cellular senescence in lung disease pathogenesis and progression. Key among these are: (i) the dynamic crosstalk between senescent cells and the immune system, including mechanisms of immune evasion and immune-mediated clearance; (ii) cell-type-specific senescence trajectories across distinct pulmonary compartments; (iii) the reversibility and phenotypic plasticity of senescent states under varying physiological and pathological conditions; and (iv) the contribution of biomechanical stressors such as altered matrix stiffness and disrupted tissue architecture to senescence induction and maintenance. In parallel, recent advances in high-dimensional omics platforms (e.g., single-cell transcriptomics, spatial proteomics) and therapeutic innovations (e.g., senolytics, senomorphics, and nanoparticle-mediated drug delivery) are paving the way for precision-targeted interventions and the identification of lung-specific senescence biomarkers. Collectively, these developments hold promise for reshaping our understanding of senescence as both a pathogenic driver and a therapeutic target in chronic lung diseases.

### 6.1. Senescence and Immune Cell Crosstalk in the Lung

Senescent epithelial and stromal cells in the lung actively modulate the immune microenvironment by secreting SASP factors that influence both innate and adaptive immune cell function.

#### 6.1.1. Modulation of Alveolar Macrophages

Senescent epithelial and stromal cells profoundly reshape immune dynamics in the lung through the secretion of a complex SASP, comprising pro-inflammatory cytokines (e.g., IL-6, IL-8), chemokines (e.g., CCL2, CXCL1), growth factors, matrix-remodeling enzymes, and EVs. A principal target of this signaling is the alveolar macrophage, which is both recruited and reprogrammed by SASP factors into a dysfunctional or M1-like pro-inflammatory state. These macrophages exhibit impaired phagocytic capacity and increased production of inflammatory mediators, thereby establishing a self-perpetuating loop of chronic inflammation and defective resolution. This phenomenon has been demonstrated in aging lungs and KRAS-driven lung cancer models [162,236,237].

Senescent stromal cells, particularly fibroblasts and mesenchymal stromal cells, contribute to this reprogramming by secreting key SASP components such as IL-6, TNF-α, GM-CSF, and CCL2, which not only enhance macrophage recruitment but also skew their phenotype toward a pro-inflammatory state. In the context of fibrosis and lung aging, these macrophages adopt an M1-like profile that sustains inflammation and impairs tissue repair. This maladaptive feedback loop has been well documented in models of IPF and senescence-associated lung injury [238,239,240].

#### 6.1.2. Modulation of T Cells

Senescent stromal cells significantly impact adaptive immunity, particularly by modulating T cell responses. While early SASP-associated chemokines can facilitate T cell infiltration, sustained exposure to SASP factors leads to T cell dysfunction. Senescent stromal cells upregulate immune checkpoint ligands such as PD-L1 and secrete immunosuppressive cytokines including TGF-β and IL-10, which collectively suppress CD8^+^ T cell proliferation, cytotoxic activity, and promote the expansion of regulatory T cells. This immunosuppressive signaling contributes to T cell exhaustion and blunted anti-tumor responses in both chronic lung disease and malignancy settings [84,241,242,243].

In the tumor microenvironment, senescent cancer-associated fibroblasts (CAFs) have been shown to suppress T cell function through both paracrine SASP signaling and direct immune checkpoint engagement. This facilitates immune evasion and supports tumor progression [84,241,244]. Such chronic immunosuppression also impairs tissue regeneration and promotes persistent pathological remodeling in non-malignant fibrotic lung disease.

#### 6.1.3. Modulation of Neutrophils

Senescent epithelial and stromal cells are potent recruiters of neutrophils through the secretion of SASP-associated chemokines such as IL-8 (CXCL8), CXCL1, and CXCL5 [245,246]. Upon recruitment, neutrophils become hyperactivated, releasing neutrophil elastase, MMPs, and ROS, which contribute to ECM degradation and tissue injury. This cascade has been well-characterized in both ALI and chronic inflammatory models, where senescent cells amplify neutrophilic infiltration and drive progressive damage [170,247].

Recent studies further underscore this mechanism; for example, senescent fibroblasts were shown to enhance neutrophil recruitment and activation, perpetuating a feed-forward loop of inflammation and tissue destruction [248].

#### 6.1.4. The Role of Immunosenescence in Worsening Chronic Lung Disease Outcomes

Immunosenescence, It is an age-associated decline in immune competence plays a pivotal role in the progression and exacerbation of chronic lung diseases such as COPD, IPF, and post-viral lung injury [249]. This process is characterized by impaired immune surveillance, a persistent low-grade inflammatory state termed “inflammaging”, and compromised tissue repair mechanisms.

In the innate immune compartment, aging leads to functional deficits in alveolar macrophages, neutrophils, and dendritic cells. Aged alveolar macrophages exhibit impaired phagocytosis and efferocytosis, resulting in defective clearance of pathogens and apoptotic cells. These cells also adopt a pro-inflammatory phenotype, producing elevated levels of TNF-α, IL-6, and IL-1β, thereby perpetuating tissue injury and fibrosis [250]. Similarly, aged neutrophils demonstrate delayed apoptosis, enhanced degranulation, and excessive ROS production, all of which contribute to alveolar damage and ECM degradation [251,252,253].

The adaptive immune system is equally affected by aging. CD4^+^ and CD8^+^ T cells show reduced clonal diversity, increased expression of exhaustion markers such as PD-1 and KLRG1, and diminished effector cytokine secretion, leading to impaired antiviral responses and delayed resolution of inflammation [254,255]. Age-related B cell dysfunction results in decreased antibody diversity and reduced responsiveness to infections and vaccinations. Notably, immunosenescence exacerbates the SASP generated by both immune and stromal cells. This sustained inflammatory milieu fosters epithelial–mesenchymal transition (EMT), fibroblast activation, and ECM deposition key processes driving chronic inflammation and fibrosis in the lung [11,35,51,250,256].

#### 6.1.5. SASP as a Driver of Immune Dysfunction in Aging Lungs

In the aging lung, persistent activation of the SASP plays a pivotal role in disrupting immune homeostasis. SASP chemokines actively recruit innate immune cells such as neutrophils and monocytes; however, the chronic inflammatory environment they establish often skews these cells toward dysfunctional or even senescent states. For instance, alveolar macrophages in aged lungs exhibit impaired phagocytic function and elevated production of inflammatory cytokines, exacerbating tissue damage rather than facilitating resolution [250].

In parallel, SASP components negatively impact adaptive immunity by impairing T cell activation, survival, and effector function. This occurs through the upregulation of immune checkpoint molecules (e.g., PD-L1) and the enrichment of immunosuppressive cytokines, ultimately promoting T cell exhaustion and blunted antiviral responses in older individuals [241,257].

Furthermore, SASP factors compromise epithelial barrier integrity and disrupt tissue repair mechanisms, facilitating increased neutrophilic infiltration and perpetuating local inflammation. This sustained, low-grade inflammatory state termed “inflammaging” has been implicated in the pathogenesis and progression of several age-related pulmonary diseases, including COPD, IPF, and post-infectious fibrotic remodeling [253,258,259]. Collectively, these effects position the SASP as a central driver of immune dysregulation in the aging lung, transforming a homeostatic and regenerative immune landscape into one marked by chronic inflammation, impaired resolution, and progressive tissue dysfunction.

### 6.2. Senescence Heterogeneity and Senescence States Across Lung Cell Types

Senescence in the lung represents a highly heterogeneous phenomenon, with distinct molecular signatures, functional consequences, and SASP profiles emerging across diverse cell types. Single-cell transcriptomic analyses have demonstrated that lung epithelial cells, endothelial cells, fibroblasts, and immune cells each adopt unique senescence programs, shaped by their specialized functions and stress responses within the tissue microenvironment [53,98,260]. The advent of high-throughput single-cell and single-nucleus RNA sequencing has enabled unprecedented resolution in characterizing these senescent states and identifying novel cell-type-specific markers [52].

For instance, using high-content imaging, Neri et al. examined senescence marker expression in primary human endothelial cells and fibroblasts and identified that G2-arrested senescent cells exhibited higher expression of canonical markers, greater IL-6 secretion, and increased sensitivity to the senolytic ABT263, compared to G1-arrested counterparts [97]. Functionally, senescent alveolar epithelial cells impair barrier integrity and reduce surfactant production, whereas senescent endothelial cells contribute to vascular permeability and leukocyte extravasation through pro-inflammatory SASP activity [55,261,262,263]. Senescent fibroblasts frequently adopt a pro-fibrotic phenotype characterized by secretion of TGF-β, IL-6, and ECM proteins that promote fibrogenesis [264].

Beyond intercellular differences, intra-lineage heterogeneity has also been documented. Single-cell analyses utilizing tools such as SenePy have revealed that even within the same cell type, distinct senescent “states” exist, each defined by variable transcriptional outputs and responses to stress stimuli [53,99]. These differences are influenced by the nature of the senescence-inducing insult whether DNA damage, oxidative stress, or oncogenic activation each eliciting a context-specific senescence program. Proteomic and transcriptomic profiling confirm that the SASP composition is not only cell-type specific but also inducer-dependent, with senescent fibroblasts producing a cytokine and matrix-modifying profile distinct from that of senescent epithelial or endothelial cells [100]. Similarly, senescence states (see above section) of the lung cells based on stimuli, type of cells, and transitory cell phenotypes may play an important role in senotyping of lung cells.

To better navigate this complexity, transcriptional marker panels such as SenMayo have been developed to detect senescent cells across multiple lung cell populations by targeting conserved features of the senescence program. Nonetheless, expression of individual markers, such as CDKN2A or p21^Cip1/Waf1^, can still vary substantially based on both cell lineage and senescence trigger [265]. Collectively, these findings emphasize that lung cellular senescence is a non-uniform, context-dependent process that requires precise, cell-type aware identification and interpretation for both mechanistic studies and the development of targeted senotherapeutics.

#### 6.2.1. Fibroblasts, Endothelial Cells, Pericytes, and Club Cells May Undergo Functionally Distinct Senescence

Senescence manifests in a functionally distinct and cell-type specific manner across various structural and stromal compartments of the lung, contributing to the overall heterogeneity of the senescent landscape. Among these, fibroblasts are well-characterized for acquiring a pro-fibrotic and pro-inflammatory phenotype upon senescence. This phenotype is typified by elevated secretion of TGF-β, IL-6, and MMPs, which not only reinforce fibrotic matrix deposition but also influence immune cell recruitment and ECM remodeling processes prominently observed in IPF [266].

In contrast, senescent endothelial cells are primarily implicated in the disruption of vascular homeostasis. They promote vascular permeability, rarefaction, and leukocyte adhesion through a distinct SASP profile enriched in chemokines such as CXCL1 and adhesion molecules like ICAM-1, collectively contributing to endothelial dysfunction and inflammation [267].

Pericytes, although less extensively studied, have been shown to undergo senescence in response to oxidative and mechanical stress. This impairs their ability to maintain capillary integrity, destabilizes endothelial–pericyte interactions, and promotes vascular leakage, thereby exacerbating pulmonary hypertension and microvascular injury [268].

Club cells, the non-ciliated epithelial progenitors residing in the bronchioles, represent another important but underappreciated senescent population. Following environmental injury or viral infection, club cells may enter a senescent state characterized by loss of proliferative capacity and lack of regenerative potential. These senescent club cells secrete cytokines such as IL-33 and amphiregulin, which alter epithelial–mesenchymal signaling and contribute to chronic airway remodeling [269]. These cell-type-specific senescence responses underscore the complexity and functional diversity of senescent cell populations in the lung, each shaping disease pathogenesis through distinct mechanisms and SASP profiles.

#### 6.2.2. Mapping Senescent Cell Subtypes with Single-Cell RNA-seq or Spatial Transcriptomics

Recent advances in single-cell RNA sequencing (scRNA-seq) and spatial transcriptomics have profoundly enhanced our understanding of senescence heterogeneity in the lung, enabling high-resolution mapping of senescent cell subtypes within their native tissue microenvironments. A landmark single-cell atlas of the aging lung revealed that senescence-associated transcriptional changes are strikingly cell-type specific, with alveolar macrophages and monocytes displaying the most pronounced alterations, while epithelial and endothelial cells followed distinct senescence trajectories [270,271]. In aged, influenza-infected mouse lungs, integrated scRNA-seq and spatial transcriptomics approaches identified spatial clustering of senescent cells particularly within fibrotic and peribronchial regions and uncovered cell-type-specific SASP signatures, including CXCL1 secretion by senescent endothelial cells [272].

The SenePy platform, applied to both human and murine lung datasets, identified type II alveolar epithelial cells and fibroblasts as key senescent populations in aged lungs, each expressing distinct senescence-associated genes such as CDKN1A and GDF15 [53]. SenMayo, a robust transcriptomic senescence signature platform, derived from human bulk RNA-seq data, has demonstrated cross-tissue applicability, facilitating senescent cell identification in the lung and other aging organs [265]. To overcome challenges in detecting senescence across diverse biological settings, the human universal senescence index (hUSI) was developed. This integrative framework enables robust identification of senescent states across aging, COVID-19, and cancer by leveraging large-scale transcriptomic datasets and highlights prognostic senescence-linked pathways [273].

Further, scDOT, a multimodal integrative tool, links cellular transcriptomes to spatial coordinates in lung tissues. When applied to human IPF samples, it revealed distinct senescent fibroblast niches and their interactions with neighboring immune and epithelial cells [274]. In parallel, SenCID, a machine learning based classification framework, was trained on 602 samples from 52 senescence transcriptome datasets spanning 30 cell types. SenCID categorizes senescent cells into six major senescence identities (SIDs), enabling reconstruction of dynamic senescence trajectories across contexts such as physiological aging, chronic lung diseases, and SARS-CoV-2 infection [99].

### 6.3. Reversibility of Senescence and Plasticity

While cellular senescence has long been regarded as a terminal, irreversible growth arrest, emerging evidence challenges this notion by revealing that certain senescent states can, under specific conditions, be at least partially reversible. This plasticity appears to be influenced by factors including cell type, the nature and severity of the inducing stressor, the duration of senescence, and the metabolic and epigenetic landscape of the cell.

Experimental studies have shown that transient senescence, particularly in response to sublethal DNA damage or oxidative stress, may be reversible. For example, oxidative stress-induced senescence in lung epithelial cells and hepatic stellate cells can be reversed upon removal of the insult or through modulation of the p53/p21^Cip1/Waf1^ pathway [93]. Similarly, mesenchymal stem cells exposed to reversible senescence stimuli demonstrate restored proliferative capacity when treated with epigenetic modulators [275].

At the molecular level, senescence reversal is associated with restoration of mitochondrial function, metabolic reprogramming, and epigenetic remodeling. Specifically, replenishment of NAD^+^ levels or improvement in mitochondrial health has been shown to suppress SASP expression and restore proliferative competence in certain senescent cell types [276]. These findings underscore that not all senescent states represent a permanent cell fate, and that therapeutic interventions aimed at modulating or reversing early or context-dependent senescence may hold promise in treating fibrosis, cancer, and age-associated dysfunction.

#### Pseudosenescence: Senescence Marker Expression Without Stable Growth Arrest

The concept of pseudosenescence refers to a transient, senescence-like cellular state in which cells express canonical markers such as p16^Ink4a^, p21^Cip1/Waf1^, and SA-β-gal activity without undergoing irreversible growth arrest. Unlike bona fide senescent cells, pseudosenescent cells retain the capacity to re-enter the cell cycle, particularly upon withdrawal of the inducing stimulus or in response to specific microenvironmental signals.

This phenomenon has been observed in epithelial progenitor cells, hematopoietic stem cells, and immune cells, particularly during tissue regeneration or repair processes [24]. For instance, during wound healing, transient expression of p21^Cip1/Waf1^ and selected SASP components facilitates a regenerative inflammatory response, which resolves as progenitor cells resume proliferation [277].

Pseudosenescence is also implicated in TIS. Tumor cells exposed to sublethal doses of radiation or chemotherapy may transiently acquire a senescent-like phenotype but subsequently escape arrest, often exhibiting increased malignancy and therapy resistance [278]. This reversibility complicates therapeutic targeting, as pseudosenescent cells may evade senolytic strategies designed to eliminate truly senescent populations. Importantly, pseudosenescence underscores the limitations of relying solely on marker-based definitions of senescence. Functional criteria such as stable growth arrest, irreversible chromatin remodeling (e.g., SAHF formation), and resistance to mitogenic signals are essential to distinguish true senescence from temporary or adaptive phenotypes.

### 6.4. Role of Mechanical Stress and ECM Stiffness

Changes in the biomechanical properties of the ECM particularly increased stiffness in fibrotic lungs and loss of structural integrity in emphysematous tissue play a critical role in both initiating and reinforcing cellular senescence. In pulmonary fibrosis, excessive deposition of collagen and cross-linked ECM proteins leads to increased tissue stiffness, which exerts abnormal mechanical stress on resident fibroblasts, epithelial cells, and endothelial cells. This mechanical cue is transduced via integrins, focal adhesion kinase (FAK), and the YAP/TAZ signaling axis, culminating in senescence-associated growth arrest and the upregulation of SASP components [279]. Fibroblasts cultured on stiff substrates or isolated from fibrotic lung tissue exhibit a robust senescent phenotype, marked by increased expression of p16^Ink4a^, IL-6, TGF-β, and matrix metalloproteinases, thereby further promoting ECM remodeling and fibrosis [280].

In contrast, emphysematous lungs are characterized by progressive ECM degradation particularly the loss of elastin and collagen resulting in weakened alveolar architecture and aberrant cell–ECM interactions. This destabilized mechanical environment induces abnormal cellular stretching, mitochondrial dysfunction, and oxidative stress, collectively driving epithelial cell senescence and impairing regenerative capacity [281]. Importantly, senescent cells within these mechanically altered tissues demonstrate enhanced resistance to apoptosis and maintain persistent SASP activity, establishing a self-perpetuating loop of matrix remodeling, inflammation, and senescence [35].

These findings emphasize that ECM mechanics are not mere consequences of chronic lung pathology, but active determinants of senescence induction and maintenance. Targeting mechanotransduction pathways or ECM remodeling processes offers a promising avenue for senescence-modulating therapies in fibrotic and degenerative lung diseases.

### 6.5. Senescence-Associated Metabolic Reprogramming

Cellular senescence in the lung is intimately associated with profound metabolic reprogramming that encompasses alterations in glycolysis, lipid metabolism, and mitochondrial bioenergetics. These metabolic shifts not only accompany the senescent phenotype but actively contribute to its initiation, maintenance, and pathological effects.

A prominent metabolic hallmark of senescent cells is a shift toward enhanced glycolysis often described as a “Warburg-like” effect even in the absence of proliferation. In senescent lung fibroblasts and epithelial cells, glycolytic reprogramming supports increased NADPH production and fuels the biosynthetic and pro-inflammatory output of the SASP [282]. In parallel, senescent cells exhibit dysregulated lipid metabolism, characterized by the accumulation of intracellular lipid droplets and upregulation of lipogenic enzymes such as fatty acid synthase. These changes are particularly evident in AT2 epithelial cells and fibroblasts from aged or fibrotic lungs, where altered lipid handling contributes to lipotoxic stress, mitochondrial dysfunction, and fibrotic activation [283,284,285].

At the mitochondrial level, senescent lung cells display altered oxidative phosphorylation (OXPHOS), elevated ROS generation, and disrupted mitochondrial dynamics, including increased fission and reduced mitophagy. These mitochondrial defects exacerbate the DNA damage response and reinforce senescence-associated growth arrest [286]. Furthermore, mitochondrial dysfunction amplifies SASP output through NF-κB activation and inflammasome signaling, linking bioenergetic collapse to persistent inflammation [20].

Importantly, interventions that target mitochondrial regulators such as PGC-1α or enhance mitophagy have been shown to attenuate senescence phenotypes and restore epithelial homeostasis in preclinical lung models [287,288]. These findings suggest that the metabolic rewiring of senescent cells is not merely epiphenomenal but represents a core driver of lung aging and disease progression.

### 6.6. Potential Target for Therapy: Metabolic Senolytics

Recent studies have elucidated distinct metabolic dependencies in senescent cells, paving the way for the development of metabolic senolytics agents that selectively eliminate senescent cells by exploiting their altered bioenergetic state. Senescent lung cells exhibit a unique metabolic phenotype characterized by enhanced glycolysis, impaired mitochondrial oxidative phosphorylation (OXPHOS), elevated ROS production, and reliance on specific survival pathways [30,286,289,290]. These adaptations render senescent cells particularly vulnerable to therapeutic strategies that disrupt redox homeostasis, NAD^+^ metabolism, or mitochondrial function.

One of the most extensively studied metabolic senolytics is navitoclax (ABT-263), a BCL-2/BCL-xL inhibitor that induces apoptosis preferentially in senescent cells by targeting their heightened mitochondrial priming [211]. Similarly, the glycolytic inhibitor 2-deoxy-D-glucose (2-DG) has demonstrated selective cytotoxicity against senescent cells with elevated glycolytic flux [291]. In preclinical lung models, pharmacologic modulation of mitochondrial health using agents such as metformin or mitophagy enhancers like urolithin A has successfully reduced senescent cell burden and mitigated fibrotic remodeling [292].

Additionally, peptide-based approaches targeting senescence-specific survival pathways have shown promise. For instance, the FOXO4-DRI peptide disrupts the interaction between p53 and FOXO4, a critical axis for senescent cell survival leading to selective apoptosis of senescent cells, including in lung tissues [215]. These findings underscore the therapeutic potential of metabolic senolytics in treating chronic lung diseases such as COPD, IPF, and post-COVID-19 pulmonary fibrosis, conditions where persistent senescent cells perpetuate inflammation and tissue remodeling. Future strategies that combine metabolic senolytics with anti-fibrotic or pro-regenerative therapies may yield synergistic effects, restoring lung homeostasis and improving clinical outcomes.

## 7. Emerging Directions and Unmet Needs

The integration of artificial intelligence (AI)-based histological analysis and machine learning (ML) algorithms is emerging as a transformative strategy for the detection and spatial characterization of senescent cells within complex tissues such as the lung. Conventional senescence markers including p16^Ink4a^, p21^Cip1/Waf1^, and SA-β-gal suffer from limited specificity and are challenging to interpret reliably in heterogeneous tissue environments. To address these limitations, AI-driven image analysis platforms, particularly deep learning-based convolutional neural networks (CNNs), have been utilized to detect senescence-associated morphological features such as cellular enlargement, nuclear irregularities, and lipofuscin accumulation directly from histological and immunofluorescence slides [293,294].

For instance, AI-assisted digital pathology has been employed to distinguish senescent fibroblasts in fibrotic lung tissues by integrating shape and texture-based descriptors with immunostaining for senescence markers [295]. Parallel advances in ML applications to single-cell RNA sequencing (scRNA-seq) and spatial transcriptomics have enabled the identification of senescent subpopulations based on transcriptional signatures, including upregulation of CDKN2A, GDF15, and key SASP components across diverse lung cell types [294].

Integrated platforms such as SenNet are being developed to combine histological features, spatial transcriptomic profiles, and imaging-derived markers, thereby enhancing the predictive power and resolution of in vivo senescence detection [51]. These approaches not only improve diagnostic capabilities but also offer tools for real-time monitoring of senotherapeutic efficacy in both preclinical models and clinical settings. Collectively, AI- and ML-based technologies represent a critical advancement in overcoming current biomarker limitations and hold significant promise for accelerating the translation of senescence research into precision pulmonary medicine.

### 7.1. Personalized Medicine: Identifying Senescent Cell Burden in Individual Patients

The concept of personalized medicine in the context of lung senescence seeks to tailor therapeutic strategies according to an individual’s senescent cell burden, spatial distribution, and cell type-specific senescence profiles. Senescent cells accumulate heterogeneously across lung compartments including alveolar epithelium, fibroblasts, endothelium, and immune infiltrates with this variation shaped by intrinsic factors such as aging and extrinsic stressors like environmental pollutants, viral infections, and fibrotic remodeling [296]. However, the clinical implementation of senescence profiling remains limited by the absence of validated in vivo biomarkers.

Transcriptomic tools such as SenMayo and SenePy developed from bulk and single-cell RNA sequencing datasets have shown promise in estimating senescent cell signatures from biopsy-derived samples [53,265]. Additional modalities, including machine learning-based histological classifiers, circulating SASP factors, and senescence-associated EVs, are under active investigation as non-invasive surrogates of senescence burden [297].

The integration of these approaches with clinical and molecular disease staging holds potential for stratifying patients into senescence-enriched or senescence-low subgroups. For example, in IPF, profiling patients with elevated senescent fibroblast signatures could guide the selective use of senotherapeutic agents alongside existing antifibrotic therapies [39]. Such personalized approaches may optimize treatment efficacy, minimize off-target toxicity, and enable longitudinal monitoring of therapeutic response.

### 7.2. Role of Environmental Pollutants, Microbiome, and Circadian Rhythms in Lung Senescence

Senescence in the lung is intricately influenced by environmental exposures, microbiome dynamics, and circadian rhythm regulation, each acting as a modifiable contributor to senescence induction and progression in chronic respiratory diseases.

Environmental pollutants such as cigarette smoke, particulate matter (PM2.5), ozone, and diesel exhaust are well-established inducers of senescence, triggering oxidative stress, DNA damage, and mitochondrial dysfunction in epithelial cells, fibroblasts, and endothelial cells [298,299,300]. For instance, cigarette smoke induces premature senescence through the activation of p16^Ink4a^ and p21^Cip1/Waf1^, alongside a pro-inflammatory SASP that perpetuates tissue injury and inflammation in COPD [40].

The lung microbiome is another key regulator. Dysbiosis marked by reduced microbial diversity and increased abundance of pro-inflammatory mediators can amplify alveolar macrophage activation and epithelial dysfunction, fueling chronic inflammation and promoting senescence via low-grade immune activation [301]. Microbial metabolites such as short-chain fatty acids (SCFAs) and lipopolysaccharides (LPS) may further influence SASP output in a context-dependent manner [302].

Circadian rhythm disruption, common in aging, shift work, or chronic stress, also contributes to lung senescence. Core clock genes (e.g., BMAL1, CLOCK, Nrf2) regulate redox homeostasis, mitochondrial health, and DNA repair. Their dysregulation has been linked to impaired alveolar regeneration and increased susceptibility to fibrotic remodeling via enhanced senescence and inflammatory signaling [303,304,305]. Thus, this highlights the importance of incorporating exposome and chrono-biological context into senescence-related research and therapeutic design.

### 7.3. Clinical Translation of Senescence-Targeted Therapies: Challenges and Progresses

Although senescence presents a promising therapeutic target in chronic lung diseases, its clinical translation is hindered by several unresolved challenges. A primary obstacle is the lack of reliable, non-invasive biomarkers for senescence in human lungs, limiting patient stratification, therapeutic monitoring, and outcome assessment [1]. Moreover, the cellular heterogeneity of senescence across lung compartments necessitates cell type- and context-specific interventions, as no universal senolytic agent is effective across all senescent cell types [7].

Current senolytic agents such as navitoclax (ABT-263) exhibit significant off-target toxicity, particularly hematologic effects, posing risks for elderly patients and those with comorbidities [306]. These safety concerns have constrained the advancement of senolytics in clinical settings.

Nevertheless, promising advances are underway. Early-phase clinical trials of dasatinib+quercetin (D+Q) have shown improved physical performance and reduced circulating SASP components in IPF patients [307]. Other compounds under development include UBX1325 (a BCL-xL inhibitor) and FOXO4-DRI peptides, which target senescent cells in fibrotic and post-COVID lung injury models. Concurrently, AI-driven histopathological scoring systems and integrative multi-omics approaches are being tested to stratify patients and predict treatment responses [308,309].

Moving forward, success in this field will rely on: Development of lung-specific senescence biomarkers; Optimization of inhalable senolytics for localized delivery; Advanced patient stratification based on molecular senescence subtypes; Long-term safety profiling and regulatory oversight to mitigate off-target risks. In sum, while hurdles persist, the clinical translation of senotherapeutics in lung disease is entering a promising phase-I, driven by molecular precision, computational insight, and a growing appreciation for the complex biology of senescence.

## 8. Future Perspectives

The expanding evidence base for senotherapeutics underscores their potential in diverse chronic lung diseases. While preclinical models and early human studies suggest efficacy, translating these findings into durable clinical benefit requires targeted strategies adapted to the unique pathobiology of each condition.

In COPD, the persistence of senescent epithelial and immune cells contributes to chronic inflammation and impaired repair. Recent work has demonstrated that the senolytic combination of dasatinib and quercetin (D+Q) can reduce senescence markers and SASP-related cytokines in both patient-derived epithelial cells and smoke-exposure mouse models, leading to broad reductions in inflammation [310]. These findings suggest that selective clearance of senescent epithelial cells may represent a disease-modifying intervention in COPD, particularly if combined with existing anti-inflammatory strategies.

For IPF, senescence of alveolar epithelial and fibroblast populations has been highlighted as a key driver of fibrotic progression. Clinical pilot trials of intermittent D+Q administration in IPF patients have demonstrated feasibility and tolerability, supporting larger-scale trials aimed at assessing efficacy [307]. Complementary systems-level analyses further suggest that senescence-associated gene signatures can define prognostic IPF subtypes, identifying high-risk groups that may benefit from more aggressive senotherapeutic interventions [311]. The integration of biomarker-driven patient stratification with targeted senotherapies could therefore help personalize therapy and improve outcomes.

Emerging drug discovery efforts are expanding beyond established senolytic regimens. Screening approaches in senescent lung fibroblasts have identified new combinations, such as dasatinib with ellagic acid or resveratrol, that show comparable or greater senolytic activity than dasatinib plus quercetin in vitro, with reduced inflammatory cytokine release during senolysis [312]. These findings expand the pharmacological toolbox and suggest that novel combinations may balance efficacy with reduced toxicity.

In parallel, repurposing drugs for metabolic diseases as senotherapeutics represents a pragmatic strategy. Observational studies and experimental models suggest that agents such as metformin and statins not only improve metabolic comorbidities but also directly attenuate lung aging processes. Importantly, alternative delivery methods such as inhalation could maximize local efficacy in the lungs while minimizing systemic adverse effects [313]. Such approaches may accelerate clinical translation by leveraging existing safety data and real-world usage patterns.

Taken together, these advances point toward a future in which senolytics and senomorphics are incorporated into a precision medicine framework for chronic lung diseases based on cellular senescence heterogeneity and states of senescence. Combining biomarker-guided patient stratification, novel senolytic combinations, and repurposed drugs with optimized delivery systems may enable disease-modifying therapies for COPD, IPF, and related conditions. Continued integration of mechanistic insights with carefully designed clinical trials will be critical to determine how these strategies can be deployed to alter the natural history of age-related lung diseases based on specific type of senescence, senescence stages/states, cell-specific senescence.

## 9. Conclusions

Cellular senescence exerts a paradoxical influence on lung health, functioning as both a protective mechanism and a contributor to lung development and disease pathogenesis. Transient or acute senescence plays a beneficial role by limiting tissue injury, facilitating wound repair, and suppressing tumorigenesis. In contrast, the chronic persistence of senescent cells is increasingly implicated in driving sustained inflammation, fibrotic remodeling, and impaired tissue regeneration across various pulmonary disorders. However, early lung development can have a contrary but transient role of cellular senescence, which needs to be studied. This dual nature highlights the necessity of delineating the contextual, temporal, and cell type specific dynamics of the senescence response.

Despite substantial progress in the field, several pivotal knowledge gaps remain. These include the absence of reliable in vivo biomarkers to track senescent cell populations, limited understanding of the reversibility of senescence and their cellular heterogeneity, and an incomplete characterization of senescent cell subsets within distinct lung compartments. Advances in high-resolution technologies such as single-cell RNA sequencing, spatial transcriptomics, metabolomic profiling, and artificial intelligence-enhanced histopathological analysis are beginning to unravel the complexity and plasticity of senescence phenotypes in the lung microenvironment.

Collectively, these insights pave the way for the development of therapeutic strategies aimed at modulating senescence. Approaches such as targeted senolytics, suppression of the senescence-associated secretory phenotype, senotyping, metabolic reprogramming, and enhancement of immune-mediated senescent cell clearance hold considerable promise for restoring lung homeostasis. Such interventions may offer clinical benefits in conditions including COPD, IPF, and post-viral fibrotic remodeling. Realizing this potential within the framework of precision medicine will require the integration of molecular profiling, rational drug design, and individualized therapeutic approaches in lung diseases associated with aging.

## Figures and Tables

**Table 1:** Regulation of Lung Cellular Heterogeneity and Senescence markers in different lung diseases.

Human/Mouse	Tissue	Marker	Cell	Disease	Hallmark	Upregulated/ Downregulated Protein	References
Mouse	Lungs	TNF	Not available	IPF	SASP	upregulated	[35]
Human	Lungs	MMP12	Not available	IPF	SASP	upregulated	[35]
Mouse	Lungs	Bcl-2	Fibroblast	IPF	SASP	downregulated	[147]
Mouse	Lungs	TGFB1	Pulmonary fibroblasts	IPF	SASP	upregulated	[35,182,183]
Human	Lungs	TNF	Not available	IPF	SASP	upregulated	[35]
Mouse	Lungs	IL1A	Epithelial cells	IPF	SASP	upregulated	[183]
Human	Lungs	TGFB1/2	Not available	IPF	SASP	downregulated	[35]
Mouse	Lungs	SERPINE1	Not available	IPF	SASP	upregulated	[35,94]
				**COPD**			
Human	Lungs	IL-6	Not available	COPD	SASP	upregulated	[184,185]
Human	Lungs	IL-10	Not available	COPD	SASP	upregulated	[184,186]
Human	Lungs	VEGFA	Not available	COPD	SASP	upregulated	[185]
Human	Lungs	IL-1B	Not available	COPD	SASP	upregulated	[184]
Human	Lungs	MMP8	Not available	COPD	SASP	upregulated	[187]
Human	Lungs	CXCL8	Not available	COPD	SASP	upregulated	[185,187]
Human	Lungs	SIRT1	Macrophages and Epithelial cells	COPD	SASP	downregulated	[137,138]
				**Mixed Diseases/Experimental Conditions**			
Mouse	Lungs	TP53	Not available	Aging, ARDS, IPF, COPD	Cell cycle arrest	upregulated	[83,86,94,131,182,183]
Mouse	Lungs	CDKN1A	Not available	Aging, Pulmonary fibrosis, ARDS, COPD	Cell cycle arrest	upregulated	[13,29,33,83,86,94,131,183,188,189]
Human	Lungs	H2AX	Not available	Aging, COPD	Other	upregulated	[110]
Mouse	Lungs	CDKN2A	Not available	Aging, emphysema	Cell cycle arrest	upregulated	[82,83,133,183,190]
Human	Lungs	GDF15	Not available	Aging, ILD, COPD, IPF	SASP	upregulated	[191,192,193]
Mouse	Lungs	H2AX	Not available	Development, Aging, IPF	DDR	upregulated	[33]
Mouse	Lungs	TNFRSF1B		Hyperoxia	SASP	upregulated	[33]
Mouse	Lungs	MMP12	Not available	IPF, CS exposure	SASP	upregulated	[35,83]
Mouse	Lungs	IL-6	Not available	IPF, CS exposure	SASP	upregulated	[35,83,194],
Human	Lungs	CDKN1A	Epithelial cells	ARDS, IPF, COPD	Cell cycle arrest	upregulated	[35,86]
Mouse	Lungs	GDF15	Not available	Pulmonary Emphysema, IPF	SASP	upregulated	[191,193,195]
Mouse	Lungs	IL-1A	Not available	IPF, Tobacco/cigarette smoke (CS) exposure	SASP	upregulated	[35,83,194]
Human	Lungs	CDKN2A	Not available	Non-small cell lung, IPF	Cell cycle arrest	upregulated	[196,197],
				**Aging/ARDS**			
Human	Lungs	TP53	Fibroblast	ARDS	Cell cycle arrest	upregulated	[35,86]
Human	Lungs	SERPINE1		Aging	SASP	upregulated	[198]
Mouse	Lungs	IL-10	Immune cells and alveolar epithelial cells	Aging	SASP	upregulated	[199]

CS: cigarette smoke, ARDS: Acute Respiratory Distress Syndrome, COPD: Chronic Obstructive Pulmonary Disease, IPF: Idiopathic Pulmonary Fibrosis.

**Table 2:** Selected senolytic compounds, their classes, mechanisms of action, and primary cellular targets.

Class	Compound	Primary Target
Tyrosine Kinase Inhibitor	Dasatinib	Tyrosine kinases, SCAP survival pathways
Flavonoid	Quercetin	PI3K pathway, mitochondria (via Bcl-2 family)
	Fisetin	PI3K/AKT pathway, mitochondria
Bcl-2 Family Inhibitor	Navitoclax (ABT-263)	Mitochondrial apoptosis regulators (Bcl-2 family)
Bcl-xL Selective Inhibitor	A1331852/A1155463	Mitochondrial apoptosis pathway (Bcl-xL)
HSP90 Inhibitor	17-DMAG/17-AAG/Geldanamycin	Chaperone protein complexes (HSP90-AKT axis)
Natural Product	Gingerenone A	Mitochondria, caspase cascade
Peptide	FOXO4-DRI	Nucleus (p53 signaling)
Immunotherapy	uPAR-targeted CAR T Cells	Cell surface receptor (uPAR)
Cardiac Glycoside	Ouabain/Digoxin	Plasma membrane (Na^+^/K^+^-ATPase pump)

Note: Senolytics are agents that selectively induce apoptosis in senescent cells by targeting senescent cell anti-apoptotic pathways (SCAPs). This table summarizes major classes of senolytics, including both natural and synthetic compounds, their known mechanisms of action, and primary molecular or organelle-level targets. The heterogeneity of senescent cells across tissues and contexts necessitates diverse mechanisms of senolytic action, such as targeting mitochondrial apoptosis regulators (e.g., Bcl-2 family), kinase signaling pathways (e.g., PI3K/AKT), or surface proteins (e.g., uPAR). Some agents, like quercetin and fisetin, may also exhibit dual senolytic and senomorphic activity depending on the cellular context.

**Table 3:** Selected senomorphic compounds, their classes, mechanisms of action, and primary cellular targets.

Class	Compound	Primary Target
mTOR Inhibitor	Rapamycin (Sirolimus)	mTORC1 complex
SIRT1 Activator	Resveratrol	Nucleus (SIRT1, NF-κB, Nrf2)
Polyphenol	Curcumin	Nucleus (NF-κB, Nrf2)
Flavonoid	Apigenin/Kaempferol	NF-κB signaling pathway
Vitamin	Niacinamide (Vitamin B3)	Nucleus/Gene transcription
Topical Agent	Niacinamide + Hyaluronic Acid	Gene expression in skin cells
Biguanide	Metformin	AMPK, NF-κB, STAT3 pathways
Statin	Atorvastatin/Pravastatin/Simvastatin	Mitochondria, NF-κB, eNOS
JAK Inhibitor	Ruxolitinib	JAK/STAT pathway
ATM Inhibitor	KU-55933/KU-60019	DDR pathway (ATM, NF-κB)
p38 MAPK Inhibitor	SB203580/UR13756/BIRB796	p38 MAPK signaling
NF-κB Inhibitor	SR12343	NF-κB signaling pathway

Note: Senomorphics are agents that suppress or modulate the pro-inflammatory and tissue-disruptive components of the SASP without eliminating senescent cells. This table summarizes major senomorphic compounds, including natural products, repurposed drugs, and synthetic molecules. Most act by targeting signaling pathways involved in SASP transcription or translation, such as NF-κB, mTOR, JAK/STAT, and p38 MAPK. Their application may preserve beneficial senescence functions while mitigating chronic inflammation and tissue dysfunction.

**Table 4:** Ongoing and Completed Clinical Trials of Senotherapeutics in Lung Diseases.

NCT Number	Condition(s)	Intervention(s)	Status	Focus of the Study
NCT02874989	IPF	Dasatinib + Quercetin (senolytic)	Completed	Pilot trial testing intermittent senolytic dosing in IPF patients for safety, feasibility, and initial efficacy signals.
NCT01708278	COPD	Quercetin (senomorphic/antioxidant)	Completed	1-week dose-escalation study to establish safety and tolerability in COPD patients.
NCT03989271	COPD	Quercetin (senomorphic)	Completed	Placebo-controlled study evaluating long-term safety and effects on airway epithelial gene expression.
NCT06003270	COPD	Quercetin (senomorphic)	Recruiting (Phase II)	Testing whether quercetin improves inflammation and clinical outcomes in COPD patients.
NCT03651895	COPD (non-diabetic)	Metformin (senomorphic; AMPK/mTOR axis)	Active, not recruiting	Studying metformin’s effects on airway glucose and senescence-linked pathways.
NCT00414648	Lymphangioleiomyomatosis (LAM)	Sirolimus (rapamycin, senomorphic)	Completed (MILES trial)	Pivotal Phase III trial showing sirolimus stabilizes lung function in LAM patients.
NCT02432560	LAM	Sirolimus	Active	Evaluating long-term safety and durability of sirolimus treatment.
NCT03150914	LAM	Low-dose Sirolimus	Active	Testing early, long-term sirolimus to prevent disease progression in LAM.
NCT03253913	LAM	Sirolimus ± Resveratrol	Completed/Active follow-up	Focused on biomarker changes (e.g., VEGF-D) in response to sirolimus with or without resveratrol.
NCT04537299	COVID-19 (older adults, hospitalized)	Fisetin (senolytic)	Active	Assessing whether fisetin reduces complications in older adults hospitalized with COVID-19.
NCT04771611	COVID-19	Fisetin (senolytic)	Active	Evaluating fisetin’s role in reducing disease severity and complications in COVID-19.

**Table 5:** Summary of Specialized Terms Related to Cellular Senescence and Lung Diseases.

Terms	Definition
**Senescent cell (SnC)**	A cell in the senescent state.
**Senescence-associated secretory phenotype (SASP)**	The context-dependent set of cytokines, chemokines, growth factors, proteases, lipids, and extracellular vesicles secreted by senescent cells.
**Senotype**	The subtype/identity of senescence in a given context (defined by trigger, markers, and SASP profile).
**Senotyping**	Experimental characterization of a senotype.
**Replicative senescence (RS)**	Senescence triggered by telomere attrition after repeated divisions, engaging DNA damage checkpoints.
**Stress-induced premature senescence (SIPS)**	Senescence caused by non-telomeric stressors (e.g., oxidative, inflammatory, toxicant exposure).
**Oncogene-induced senescence (OIS)**	Senescence elicited by oncogene hyperactivation as an early tumor-suppressive barrier.
**Therapy-induced senescence (TIS)**	Senescence induced by chemo- or radiotherapy in normal or malignant cells.
**Mitochondrial dysfunction-associated senescence (MiDAS)**	A senescence program driven by mitochondrial/metabolic stress with a distinct SASP signature.
**Paracrine (bystander) senescence**	Senescence induced in neighboring cells by SASP factors released from senescent cells.
**Pseudosenescence**	A reversible state that mimics some senescence markers (e.g., SA-β-gal, p16^INK4a) without establishing stable, irreversible cell-cycle arrest.
**Immunosenescence**	Age-associated decline of immune function, including impaired clearance of senescent cells.
**Inflammaging**	Chronic, low-grade sterile inflammation associated with aging and sustained SASP activity.
**Senolytics**	Agents that preferentially eliminate senescent cells.
**Senomorphics (senostatics)**	Agents that suppress deleterious SASP/signaling without killing senescent cells.
**Senotherapy/Senotherapeutics**	Umbrella term for therapeutic strategies targeting senescence (senolytics and senomorphics).
**SA-β-gal (senescence-associated β-galactosidase)**	A commonly used senescence activity readout measured at pH 6.0.
**DNA damage response (DDR)**	Checkpoint and repair signaling activated by DNA damage that helps enforce senescence.
**SAHF (senescence-associated heterochromatin foci)**	Repressive chromatin structures that stabilize proliferation arrest.
**DNA-SCARS**	DNA segments with chromatin alterations that reinforce persistent DDR signaling in senescence.
**cGAS–STING signaling**	Cytosolic DNA sensing pathway that amplifies SASP and innate immune signaling in senescence.
**YAP–TAZ mechanotransduction**	Matrix- and stiffness-responsive transcriptional regulators that can modulate fibroblast activation and senescence.
**AT2 (AEC2) and AT1 cells**	Alveolar type II progenitor cells and flattened type I gas-exchange cells of the distal lung.
**Epithelial–mesenchymal transition (EMT)**	A cell-state change linked to fibrosis and, in some contexts, senescence-associated remodeling.
**TGF-β signaling**	Profibrotic pathway frequently intersecting with senescence programs and SASP output.
**NF-κB/JAK–STAT/mTOR**	Canonical inflammatory and growth pathways that regulate SASP composition and intensity.
**p16^INK4a^**	CDK inhibitor that enforces RB-mediated cell-cycle arrest in senescence.
**p21^Cip1/Waf1^**	CDK inhibitor downstream of p53 that enforces G1/S arrest in senescence.
**TP^53^ (p53)**	Tumor-suppressor transcription factor that initiates cell-cycle arrest and senescence upon stress/DNA damage.
**Telomere attrition**	Progressive telomere shortening that precipitates replicative senescence.
**Autophagy/Mitophagy**	Catabolic pathways that remodel proteostasis and mitochondria during senescence.
**BCL-2 family dependency**	Anti-apoptotic reliance (e.g., BCL-xL, BCL-2, MCL-1) common in senescent cells and targetable by senolytics.
**Senescence escape**	Loss of stable arrest and re-entry into the cell cycle from a senescent-like state.

## Data Availability

No new data were created or analyzed in this study.

## Abbreviation

**Abbreviation**	**Full Form**
DDR	DNA Damage Response
SASP	Senescence-Associated Secretory Phenotype
COPD	Chronic Obstructive Pulmonary Disease
IPF	Idiopathic Pulmonary Fibrosis
OIS	Oncogene-Induced Senescence
TIS	Therapy-Induced Senescence
MiDAS	Mitochondrial Dysfunction-Associated Senescence
AMPK	AMP-activated Protein Kinase
AT2	Alveolar Type II Cells
ECM	Extracellular Matrix
ROS	Reactive Oxygen Species
PDGF-AA	Platelet-Derived Growth Factor-AA
MMPs	Matrix Metalloproteinases
p53	Tumor Suppressor Protein 53
p21	Cyclin-Dependent Kinase Inhibitor 1
p16	Cyclin-Dependent Kinase Inhibitor 4A
Rb	Retinoblastoma
ALI	Acute Lung Injury
ARDS	Acute Respiratory Distress Syndrome
SARS-CoV-2	Severe Acute Respiratory Syndrome Coronavirus 2
EVs	Extracellular Vesicles
rhCC16	Recombinant Human Clara Cell Protein 16
SIRT1	Sirtuin 1
PAI-1	Plasminogen Activator Inhibitor-1
TGF-β	Transforming Growth Factor Beta
CTGF	Connective Tissue Growth Factor
PDGF	Platelet-Derived Growth Factor
PAECs	Pulmonary Artery Endothelial Cells
PASMCs	Pulmonary Artery Smooth Muscle Cells
YWHAZ	Tyrosine 3-Monooxygenase/Tryptophan 5-Monooxygenase Activation Protein Zeta
ABT-737	Senolytic Therapy Drug
FOXO4-DRI	Forkhead Box O4-Drug Resistance Inhibitor
SA-β-Gal	Senescence-Associated β-Galactosidase
γH2A.X	Gamma-Histone H2A Variant X
DNAmAge	DNA Methylation Age
AgeAcc	Age Acceleration
WGCNA	Weighted Gene Co-Expression Network Analysis
IL-10	Interleukin 10
TP53	Tumor Protein 53
H2AX	H2A.X Variant Histone
CDKN2A	Cyclin-Dependent Kinase Inhibitor 2A
GDF15	Growth Differentiation Factor 15
CDKN1A	Cyclin-Dependent Kinase Inhibitor 1A
TNFRSF1B	Tumor Necrosis Factor Receptor Superfamily Member 1B
Bcl2 L1	BCL2 Like 1
CXCL8	C-X-C Motif Chemokine Ligand 8
IL1A	Interleukin 1 Alpha
MMP12	Matrix Metallopeptidase 12
SERPINE1	Serine Protease Inhibitor, Clade E, Member 1 (Plasminogen Activator Inhibitor-1)
TGFβ1	Transforming Growth Factor Beta 1
TNF	Tumor Necrosis Factor
IL-6	Interleukin 6
IL-1β	Interleukin 1 Beta
MMP-8	Matrix Metallopeptidase 8
VEGFA	Vascular Endothelial Growth Factor A
SnCs	Senescent Cells
NF-κB	Nuclear Factor Kappa B
mTOR	Mammalian Target of Rapamycin
p38 MAPK	p38 Mitogen-Activated Protein Kinase
JAK/STAT	Janus Kinase/Signal Transducer and Activator of Transcription
ATM	Ataxia Telangiectasia Mutated
STACs	Sirtuin-Activating Compounds
Nrf2	Nuclear Factor Erythroid 2-Related Factor 2
DAMPs	Damage-Associated Molecular Patterns
TAME	Targeting Aging with Metformin
eNOS	Endothelial Nitric Oxide Synthase
Ruxolitinib	JAK Inhibitor
Rapalogs	Rapamycin Analogs
SR12343	NF-κB Inhibitor
